# Stature prediction using anthropometric measurements of the hand in a sample of adult Egyptian, Arab, and Malaysian populations

**DOI:** 10.1038/s41598-025-15447-1

**Published:** 2025-09-23

**Authors:** Heba Abdel-Samie Mohamed Hussein, Omneya Ibrahim Mohamed

**Affiliations:** Forensic Medicine and Clinical Toxicology Department, Faculty of Medicine, Alexandria University, Alexandria, Egypt

**Keywords:** Biological profile, Palm, Phalanges, Height estimation, Anthropometry, Identification, Biological techniques, Medical research

## Abstract

Among the major parameters of any biological profile, stature holds particular significance in forensic investigations. It may be problematic to have the hands of the victim in an integral form at different crime scenes. This study aimed to predict stature using different hand, fingers, and phalanges measurements in a sample of Egyptians, Arabs from the Gulf area and Malaysians. The present cross-sectional study included 300 adult, male and female healthy subjects (100 Egyptians, 100 Arabs from the Gulf area, and 100 Malaysians). Measurements included hand length, hand breadth, each finger length, and each phalanx length from the right hands of the study participants using a digital vernier calliper. Significant differences among the three studied populations regarding stature, together with hand, finger, and phalanx measurements. Males had significantly higher values than females in the three studied groups (Egyptians, Arabs and Malaysians), according to stature and all studied hand measurements. The multiple regression models obtained were population- and sex-specific, and they had higher estimates of reliability than the simple regression models. It is worth noting that simple linear regression models can be more useful when only part of a damaged hand is found at the crime scene. This scenario is more common than we might think, especially in mass disasters or murders, making the research on these models highly relevant.

**Supplementary Information:**

The online version contains supplementary material available at 10.1038/s41598-025-15447-1.

## Introduction

Forensic anthropometry plays a crucial role in accurately identifying a body by formulating a biological profile from its partially damaged or dismembered parts^1^. The assessment of age, sex, geographical origin, and stature constitutes the significant parameters of any biological profile. Among these parameters, stature holds particular significance in forensic investigations due to its complicated interplay between genetics and environmental factors 2. Human stature can be verified by analysis of a combination of linear body dimensions. Understanding the relationship between specific body dimensions and proportions is of great help in criminal investigations, as it permits estimating an individual’s stature even when only a part of the body is available as evidence^2,3^.

Various techniques have emerged for stature prediction, some utilizing dry bones, which involve direct osteometric measurements of the skeletal elements after soft tissues have been removed, often requiring adjustments for post-mortem bone shrinkage. Other methods involve anthropometric measurements taken from the intact body parts of living individuals or cadavers. It’s important to note that regression models derived from either measurement are variable, as the presence or absence of soft tissue significantly alters the dimensions and, thus, the predictive models. This variability presents a challenge that keeps the research process engaging and dynamic^4–10^.

Previous research has determined stature in humans using anthropometric measurements of different body parts, like the upper extremities, lumbar vertebrae, and foot. Those measurements are specific to each population, sex, and ethnic group^11–22^. The human hand is a vital anatomical feature, and its utility in forensic identification, especially following mass disasters, is of utmost significance^23^. Most previously published research that estimated stature, using measurements of the hand, relied on both hand length and hand breadth, with limited focus on linking stature to finger and phalanx measurements^24–27^. Furthermore, scientists must use updated formulae derived for a particular population because of secular trends, which refer to the long-term changes in a population’s physical characteristics. Additionally, comparative studies should be conducted to clarify the sex and geographical origin differences among various populations.

Given the diverse population of Egypt, there is a pressing need for a dependable and straightforward technique for identifying these various populations. This is particularly crucial in the context of the Bachelor of International Medical Program (IMP) in the faculties of medicine across Egypt, which has accepted international students from Malaysia and many other Arab and African countries. Currently, the number of students registered in the IMP at the Faculty of Medicine, University of Alexandria, is approximately 2500, including students from Kuwait, Saudi Arabia, Palestine, Syria, Iraq, Jordan, Sudan, Malaysia, and other nationalities. This underscores the importance of our proposed study.

The potential impact of our findings on the field of forensic anthropology is significant, as it could provide a reliable and straightforward technique for identifying these diverse populations. Our study’s purpose is to predict stature using various hand, finger, and phalanx measurements in a sample of Egyptians, Arabs from the Gulf region, and Malaysians by developing population-specific models in a sample of individuals from Egyptian, Gulf Arab, and Malaysian populations.

## Materials and methods

### Subjects

This cross-sectional study included 300 healthy adult male and female subjects (100 Egyptians, 100 Arabs from the Gulf region, and 100 Malaysians). Each population group was equally divided into 50 males and 50 females. The mean age of male research subjects was 23.52 ± 1.53, and for female subjects was 23.27 ± 1.07, with a non-significant difference between the ages of males and females (t = 1.666 and *p* = 0.097), ensuring an unbiased and fair representation of the data. Moreover, the statistical findings of comparable age ranges between males and females in each population group are of significant importance. In the Egyptian group, the mean age of males was 23.28 ± 1.13 and females was 23.04 ± 0.88 (t = 1.174 and *p* = 0.082). In the Arab group, the mean ages of males and females were 23.96 ± 0.45 and 24.16 ± 1.92 (t = 1.802 and *p* = 0.075). In the Malaysian group, the mean ages of males and females were 23.36 ± 1.35 and 23.36 ± 1.40 (t = 0.000 and *p* = 1.000).

A statistician using G*Power software calculated the sample size for this study. The study participants were volunteer medical students from different study years at the Faculty of Medicine, Alexandria University, Egypt, with an age range of 21–27 years. Notably, participants with hand deformities, diseases, and injuries were excluded.

Each participant’s informed consent was obtained in a meticulous process that respected his or her autonomy and rights. This involved collecting demographic data and history in a simple questionnaire, which included age, sex, nationality, habits, medical and surgical history, and previous trauma in the hand. The study researchers also took the time to explain the aim and the details of the study to every participant, ensuring complete understanding and voluntary participation.

### Methods

In the present study, the stature of each participant was measured using a manual stadiometer. To take the measurement, every study participant was instructed to stand straight and without any footwear or headwear. The distance from the floor to the top of the head was determined, in centimeters, by aligning the stadiometer’s measuring rod with the top of the participant’s head, ensuring the rod was perpendicular to the floor. 

**Fig. 1:**
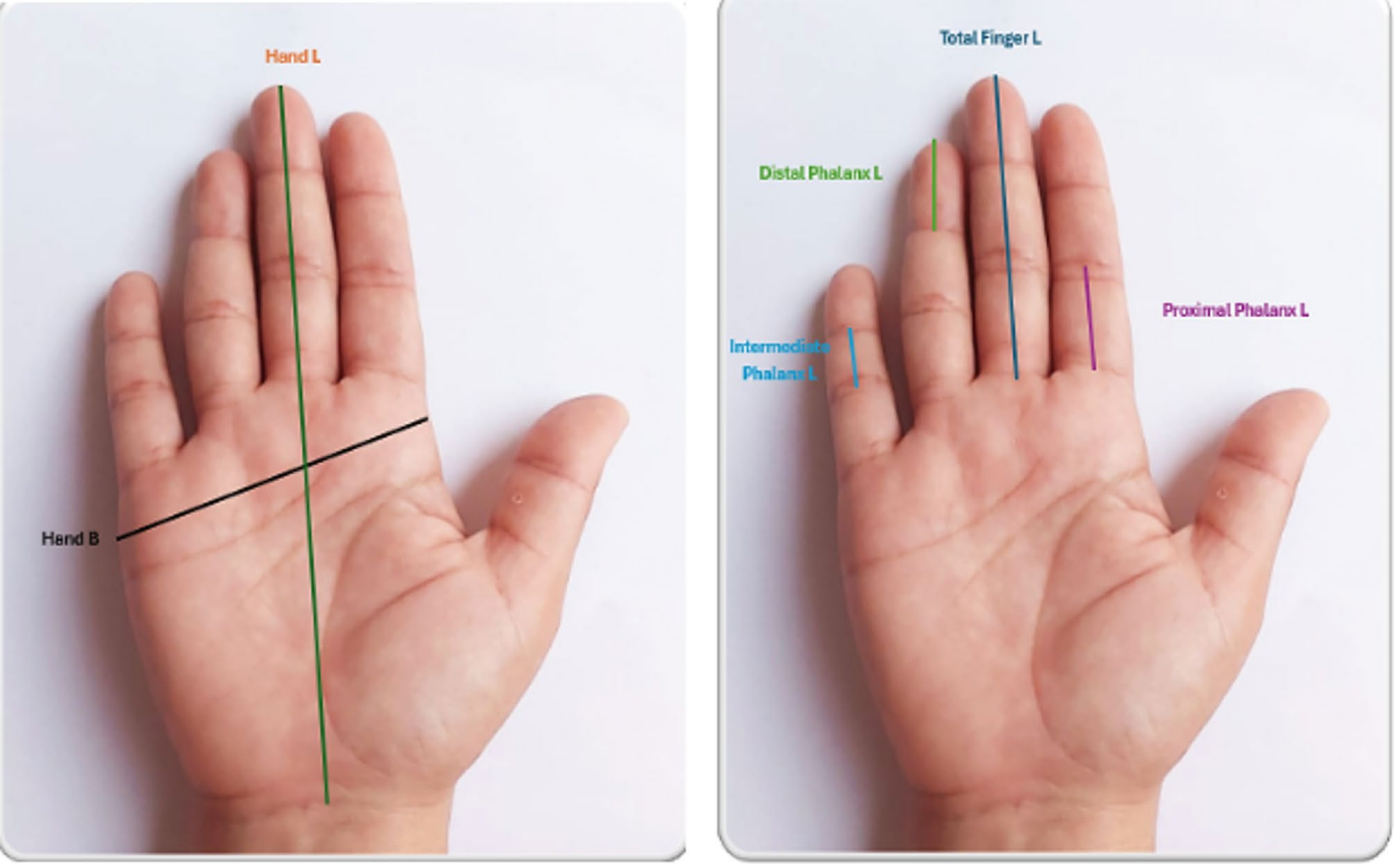
Hand measurements done in this study.

Our procedures for the anthropometric measurements of the hands were meticulously standardized to ensure the reliability of the results. These measurements, taken in centimeters using a digital vernier calliper from the right hands of the study participants, included hand length, hand breadth, the length of each finger, and phalanx length. To minimize inter-observer errors, the same researcher made all the measurements. Repeated measurements for 50 research subjects revealed an insignificant intra-observer difference (*p* > 0.05). Moreover, the intra-observer reliability was excellent (correlation coefficient = 0.90)^28^. Each study participant was asked to place their hand on a uniform horizontal surface with the extension of all fingers and abduction of the thumb finger, as shown in Fig. 1.

Hand length was measured as the distance from the mid-point of the distal crosswise crease of the wrist to the maximum anterior point of the middle finger. Handbreadth was defined as the distance from the maximal lateral point on the head of the metacarpal bone of the index finger to the maximal medial point on the head of the metacarpal bone of the little finger. At the same time, each finger’s total length (thumb, index, middle, ring, and little fingers) was measured as the distance between the tip of the finger and the border crease of the palm. Moreover, the length of each phalanx of all fingers was measured, as shown in Fig. 1.

The proximal phalanx length of the thumb, index, middle, ring, and little finger was measured as the distance from the proximal interphalangeal joint crease to the metacarpophalangeal joint crease of each finger. The index, middle, ring, and little finger intermediate phalanx length was calculated as the distance from the distal interphalangeal joint crease to the proximal interphalangeal joint crease. The thumb, index, middle, ring, and little finger distal phalanx lengths were measured as the distance from the most forward projecting point on the tip of each finger to the distal interphalangeal joint crease of each finger^29^.

### Statistical analysis

Data were fed to the computer and analysed using the IBM SPSS software package version 23.0. Qualitative data were described using numbers and percentages. The Kolmogorov-Smirnov test was used to verify the normality of the distribution. Quantitative data were described using mean and standard deviation. The significance of the results obtained was judged at the 5% level. The student t-test was used for normally distributed quantitative variables to compare between two studied groups. The Pearson coefficient was applied to correlate two normally distributed quantitative variables. The F-test (ANOVA) for normally distributed quantitative variables was used to compare more than two groups. Simple and multiple linear regression were used to estimate stature. A regression equation was developed, including the coefficients (R, R², SEE, and F), to estimate stature and body weight from different studied hand measurements, ensuring the validity of the results^30^.

### Ethical considerations

Ethical approval of the study was obtained from the Research Ethics Committee of the Faculty of Medicine, Alexandria University (IRB Number: 00012098, FWA Number: 00018699, Serial Protocol Number: 0305212). Moreover, all methods were performed following the Declaration of Helsinki.

## Results

### Descriptive analysis for stature and anthropometric measurements of the hand

#### Population differences

As shown in Table 1, the current study demonstrated a statistically significant difference among the three studied population groups according to the stature (F = 12.308, *p* < 0.001), hand length (F = 10.051, *p* < 0.001), and handbreadth (F = 5.562, *p* = 0.004). Moreover, all thumb measurements were significantly different among the three studied population groups (F = 35.399, 10.266, 15.430, and *p* < 0.001), respectively, for the total length of the distal and proximal phalanges. All index finger measurements were significantly different among the three studied populations with (F = 11.994, 4.921, 9.473, and 17.865, *p* < 0.001, 0.008, < 0.001) respectively for the total length of the distal, intermediate, and proximal phalanges of the index finger. Regarding the middle finger, the length of the intermediate and proximal phalanges of the middle finger showed statistically significant differences among the studied population groups, where (F = 10.057 and 7.317, *p* < 0.001 and 0.001) respectively. As regards the ring finger, the length of the distal, intermediate, and proximal phalanges was significantly different among the studied population groups, where (F = 4.987, 8.322 and 9.098, *p* = 0.007 and < 0.001) respectively. Furthermore, the total and the length of the proximal and distal phalanges of the little finger showed statistically significant differences among Egyptians, Arabs, and Malaysians (F = 5.633, 10.461 and 8.583, *p* = 0.004, < 0.001 and < 0.001), respectively.

**Table 1:** Descriptive analysis, according to population group, for stature (in centimetres) and anthropometric measurements of the hand (in centimetres) in a sample of adult egyptians, Arabs and malaysians. (*n* = 300)

	Egyptians (*N* = 100)	Arabs (*N* = 100)	Malaysians (*N* = 100)	F	*p*
*Stature*	169.15 ± 11.41	165.02 ± 15.95	160.62 ± 7.65	12.308*	< 0.001*
*Hand L*	18.85 ± 1.61	18.37 ± 1.71	17.88 ± 1.23	10.051*	< 0.001*
*Hand B*	8.70 ± 0.74	8.63 ± 1.25	8.28 ± 0.85	5.562*	0.004*
*Thumb*					
Total	6.90 ± 0.64	6.67 ± 0.84	6.08 ± 0.63	35.399*	< 0.001*
Distal	3.57 ± 0.56	3.52 ± 0.52	3.26 ± 0.46	10.266*	< 0.001*
Proximal	3.31 ± 0.51	3.27 ± 0.99	2.82 ± 0.47	15.430*	< 0.001*
*Index*					
Total	7.54 ± 0.65	7.19 ± 0.69	7.10 ± 0.69	11.994*	< 0.001*
Distal	2.67 ± 0.42	2.65 ± 0.37	2.52 ± 0.30	4.921*	0.008*
Intermediate	2.37 ± 0.22	2.29 ± 0.25	2.22 ± 0.23	9.473*	< 0.001*
Proximal	2.54 ± 0.37	2.24 ± 0.30	2.36 ± 0.39	17.865*	< 0.001*
*Middle*					
Total	8.22 ± 0.73	7.99 ± 0.90	8.01 ± 0.70	2.706	0.068
Distal	2.70 ± 0.41	2.71 ± 0.47	2.62 ± 0.21	1.520	0.220
Intermediate	2.81 ± 0.37	2.66 ± 0.33	2.62 ± 0.24	10.057*	< 0.001*
Proximal	2.74 ± 0.43	2.62 ± 0.34	2.83 ± 0.41	7.317*	0.001*
*Ring*					
Total	7.43 ± 0.77	7.20 ± 0.94	7.23 ± 0.63	2.546	0.080
Distal	2.62 ± 0.39	2.67 ± 0.37	2.51 ± 0.30	4.987*	0.007*
Intermediate	2.61 ± 0.44	2.45 ± 0.32	2.42 ± 0.26	8.322*	< 0.001*
Proximal	2.21 ± 0.44	2.09 ± 0.44	2.33 ± 0.30	9.098*	< 0.001*
*Little*					
Total	6.06 ± 0.77	5.95 ± 0.90	5.70 ± 0.66	5.633*	0.004*
Distal	2.40 ± 0.56	2.52 ± 0.33	2.23 ± 0.40	10.461*	< 0.001*
Intermediate	1.75 ± 0.33	1.80 ± 0.24	1.76 ± 0.28	0.989	0.373
Proximal	1.87 ± 0.46	1.63 ± 0.49	1.67 ± 0.28	8.583*	< 0.001*

F: for ANOVA test *: Statistically significant at *p* ≤ 0.05.

#### Sex differences

According to the data in Table 2, males in the present study were significantly taller than females in the three studied groups, with t = 5.968, *p* < 0.001 in Egyptians, t = 14.490, *p* < 0.001 in Arabs, and t = 9.408, *p* < 0.001 in Malaysians. Moreover, males had significantly higher values than females in the three studied groups regarding hand measurements (hand length t = 5.026, *p* < 0.001, t = 13.599, *p* < 0.001 and t = 18.997, *p* < 0.001) in the three groups, respectively. While for handbreadth, (t = 6.682, *p* < 0.001in the Egyptian group, t = 12.337, *p* < 0.001 in Arabs, t = 18.520, *p* < 0.001 in Malaysians. Furthermore, all individual finger measurements were significantly taller in males than females in the three studied groups, except for the length of the proximal phalanx of the thumb in Egyptians and Arabs groups, total length, intermediate and proximal phalanx lengths of the index and middle fingers, in Egyptians, and proximal phalanx length of the index finger in Malaysians and intermediate and proximal phalanges lengths of the ring finger in Egyptians. These exceptions, while intriguing, also highlight the need for further research to fully understand the complexities of sex differences in anthropometry.

**Table 2:** Descriptive analysis, according to sex, for stature (in centimetres) and anthropometric measurements of the hand (in centimetres) in the three studied groups; egyptians, Arabs and malaysians. (*n* = 300)

	Egyptians				Arabs				Malaysians			
	Males (*n* = 50)	Females (*n* = 50)	t	*p*	Males (*n* = 50)	Females (*n* = 50)	t	*p*	Males (*n* = 50)	Females (*n* = 50)	t	*p*
*Stature*	164.55 ± 7.2	156.69 ± 5.9	5.968*	< 0.001*	178.52 ± 5.9	159.77 ± 6.9	14.490*	< 0.001*	175.95 ± 4.3	154.09 ± 15.8	9.408*	< 0.001*
*Hand Length*	18.44 ± 1.33	17.33 ± 0.81	5.026*	< 0.001*	20.14 ± 1.04	17.56 ± 0.85	13.599*	< 0.001*	19.88 ± 0.95	16.86 ± 0.61	18.997*	< 0.001*
*Hand Breadth*	8.75 ± 0.98	7.80 ± 0.21	6.682*	< 0.001*	9.28 ± 0.43	8.13 ± 0.50	12.337*	< 0.001*	9.73 ± 0.74	7.54 ± 0.40	18.520*	< 0.001*
*Thumb*												
Total	6.28 ± 0.62	5.88 ± 0.58	3.364*	0.001*	7.34 ± 0.39	6.45 ± 0.53	9.667*	< 0.001*	7.34 ± 0.57	6.0 ± 0.43	13.299*	< 0.001*
Distal	3.47 ± 0.47	3.05 ± 0.34	5.196*	< 0.001*	3.97 ± 0.31	3.16 ± 0.45	10.309*	< 0.001*	3.95 ± 0.28	3.09 ± 0.30	14.679*	< 0.001*
Proximal	2.83 ± 0.45	2.80 ± 0.49	0.276	0.783	3.38 ± 0.59	3.24 ± 0.42	1.309	0.194	3.64 ± 1.25	2.91 ± 0.39	3.948*	< 0.001*
*Index*												
Total	7.19 ± 0.83	7.01 ± 0.51	1.265	0.210	7.96 ± 0.58	7.13 ± 0.40	8.389*	< 0.001*	7.74 ± 0.43	6.63 ± 0.39	13.384*	< 0.001*
Distal	2.65 ± 0.35	2.39 ± 0.17	4.691*	< 0.001*	2.92 ± 0.32	2.41 ± 0.36	7.544*	< 0.001*	2.98 ± 0.18	2.33 ± 0.18	18.137*	< 0.001*
Intermediate	2.21 ± 0.29	2.24 ± 0.16	0.642	0.523	2.42 ± 0.19	2.31 ± 0.23	2.518*	0.013*	2.47 ± 0.14	2.12 ± 0.22	9.586*	< 0.001*
Proximal	2.33 ± 0.44	2.39 ± 0.35	0.683	0.496	2.63 ± 0.33	2.45 ± 0.40	2.378*	0.019*	2.29 ± 0.36	2.18 ± 0.23	1.767	0.081
*Middle*												
Total	8.05 ± 0.63	7.98 ± 0.77	0.513	0.609	8.69 ± 0.48	7.75 ± 0.62	8.551*	< 0.001*	8.71 ± 0.50	7.26 ± 0.56	13.556*	< 0.001*
Distal	2.66 ± 0.27	2.58 ± 0.12	1.995*	0.049*	2.92 ± 0.35	2.47 ± 0.34	6.390*	< 0.001*	3.11 ± 0.20	2.30 ± 0.28	16.502*	< 0.001*
Intermediate	2.62 ± 0.28	2.61 ± 0.19	0.042	0.967	2.94 ± 0.41	2.68 ± 0.29	3.718*	< 0.001*	2.87 ± 0.20	2.45 ± 0.31	7.995*	< 0.001*
Proximal	2.81 ± 0.47	2.86 ± 0.35	0.626	0.533	2.90 ± 0.39	2.58 ± 0.41	3.718*	< 0.001*	2.87 ± 0.20	2.45 ± 0.31	7.995*	< 0.001*
*Ring*												
Total	7.47 ± 0.68	6.99 ± 0.46	4.195*	< 0.001*	8.03 ± 0.40	6.84 ± 0.56	12.298*	< 0.001*	7.97 ± 0.51	6.44 ± 0.57	14.233*	< 0.001*
Distal	2.67 ± 0.30	2.34 ± 0.18	6.592*	< 0.001*	2.69 ± 0.40	2.54 ± 0.38	2.009*	0.047*	2.97 ± 0.22	2.36 ± 0.21	14.161*	< 0.001*
Intermediate	2.47 ± 0.34	2.37 ± 0.15	1.842	0.070	2.94 ± 0.33	2.27 ± 0.23	11.627	< 0.001*	2.62 ± 0.22	2.27 ± 0.31	6.479*	< 0.001*
Proximal	2.33 ± 0.37	2.33 ± 0.22	0.066	0.948	2.33 ± 0.28	2.09 ± 0.53	2.895*	0.005*	2.37 ± 0.44	1.81 ± 0.18	8.491*	< 0.001*
*Little*												
Total	5.91 ± 0.78	5.48 ± 0.41	3.484*	0.001*	6.65 ± 0.52	5.46 ± 0.45	12.141*	< 0.001*	6.72 ± 0.54	5.18 ± 0.35	16.910*	< 0.001*
Distal	2.45 ± 0.44	2.02 ± 0.17	6.418*	< 0.001*	2.63 ± 0.65	2.17 ± 0.30	4.561*	< 0.001*	2.79 ± 0.19	2.24 ± 0.17	15.066*	< 0.001*
Intermediate	1.87 ± 0.31	1.64 ± 0.19	4.595*	< 0.001*	1.89 ± 0.24	1.61 ± 0.34	4.704*	< 0.001*	1.98 ± 0.13	1.63 ± 0.20	10.414*	< 0.001*
Proximal	1.59 ± 0.31	1.75 ± 0.22	3.018*	0.003*	1.98 ± 0.44	1.75 ± 0.45	2.655*	0.009*	1.95 ± 0.42	1.31 ± 0.32	8.514*	< 0.001*

t: Student t-test *: Statistically significant at *p* ≤ 0.05.

Hand L = hand length Hand B hand breadth Total = total length of the finger Distal = length of the distal phalanx of the finger.

Intermediate = length of the Intermediate phalanx of the finger Proximal = length of the proximal phalanx of the finger.

### Correlation between stature with each of the hand measurements individually in males and females in the three studied groups

#### Egyptian group

Table 3 illustrates that all the studied hand measurements showed a significant positive correlation with stature in the group of Egyptian males, with correlation coefficients ranging from 0.307 (of the proximal phalanx of the thumb) to 0.861 (of the total length of the middle finger). In the Egyptian females’ group, the hand length, the handbreadth, the length of the distal and intermediate phalanges of the index finger, the length of the intermediate phalanx of the middle finger, the length of the intermediate phalanx of the ring finger and the length of the distal phalanx of the little finger showed a significant positive correlation with stature. The correlation coefficients ranged from 0.301(for the distal phalanx of the ring finger) to 0.870 (for the intermediate phalanx of the ring finger).

**Table 3:** Correlation between stature with hand measurements individually in males and females in the three studied groups. (*n* = 300)

	Egyptians				Arabs				Malaysians			
	Males (*n* = 50)		Females (*n* = 50)		Males (*n* = 50)		Females (*n* = 50)		Males (*n* = 50)		Females (*n* = 50)	
	Stature		Stature		Stature		Stature		Stature		Stature	
	*r*	*p*	*r*	*p*	*r*	*p*	*r*	*p*	*r*	*p*	*r*	*p*
*Hand L*	0.838*	< 0.001*	0.742*	< 0.001*	0.488*	< 0.001*	0.669*	< 0.001*	0.663*	< 0.001*	0.360*	0.010*
*Hand B*	0.404*	0.004*	0.378*	0.007*	0.273	0.055	0.170	0.239	0.457*	0.001*	0.610*	< 0.001*
*Thumb*												
Total	0.673*	< 0.001*	0.652*	< 0.001*	0.483*	< 0.001*	0.268	0.060	0.171	0.236	0.036	0.804
Distal	0.560*	< 0.001*	0.002	0.991	0.095	0.513	0.082	0.573	0.592*	< 0.001*	0.126	0.385
Proximal	0.307*	0.030*	0.834*	< 0.001*	0.370*	0.008*	0.035	0.807	0.131	0.365	0.137	0.343
*Index*												
Total	0.848*	< 0.001*	0.005	0.971	0.060	0.677	0.379*	0.007*	0.698*	< 0.001*	0.247	0.083
Distal	0.676*	< 0.001*	0.659*	< 0.001*	0.044	0.762	0.386*	0.006*	0.308*	0.029*	0.712*	< 0.001*
Intermediate	0.402*	0.004*	0.319*	0.024*	0.081	0.574	0.386*	0.006*	0.655*	< 0.001*	0.319*	0.024*
Proximal	0.804*	< 0.001*	0.488*	< 0.001*	0.219	0.127	0.107	0.459	0.467*	0.001*	0.690*	< 0.001*
*Middle*												
Total	0.861*	< 0.001*	0.186	0.196	0.331*	0.019*	0.794*	< 0.001*	0.652*	< 0.001*	0.337*	0.017*
Distal	0.462*	0.001*	0.068	0.641	0.183	0.204	0.298*	0.035*	0.323*	0.022*	0.359*	0.010*
Intermediate	0.534*	< 0.001*	0.417*	0.003*	0.231	0.106	0.631*	< 0.001*	0.558*	< 0.001*	0.647*	< 0.001*
Proximal	0.776*	< 0.001*	0.079	0.584	0.420*	0.002*	0.562*	< 0.001*	0.377*	0.007*	0.406*	0.003*
*Ring*												
Total	0.775*	< 0.001*	0.197	0.170	0.549*	< 0.001*	0.430*	0.002*	0.643*	< 0.001*	0.465*	0.001*
Distal	0.463*	0.001*	0.301*	0.033*	0.760*	< 0.001*	0.340*	0.016*	0.361*	0.010*	0.352*	0.012*
Intermediate	0.495*	< 0.001*	0.870*	< 0.001*	0.333*	0.018*	0.730*	< 0.001*	0.335*	0.018*	0.633*	< 0.001*
Proximal	0.581*	< 0.001*	0.175	0.224	0.154	0.286	0.058	0.687	0.393*	0.005*	0.759*	< 0.001*
*Little*												
Total	0.768*	< 0.001*	0.468*	0.001*	0.186	0.197	0.409*	0.003*	0.595*	< 0.001*	0.633*	< 0.001*
Distal	0.653*	< 0.001*	0.350*	0.013*	0.301*	0.034*	0.038	0.795	0.354*	0.012*	0.508*	< 0.001*
Intermediate	0.560*	< 0.001*	0.038	0.796	0.123	0.393	0.316*	0.026*	0.242	0.090	0.045	0.757
Proximal	0.452*	0.001*	0.780*	< 0.001*	0.534*	< 0.001*	0.347*	0.014*	0.531*	< 0.001*	0.918*	< 0.001*

r: Pearson coefficient *: Statistically significant at *p* ≤ 0.05 Hand L = hand length Hand B hand breadth Total = total length of the finger Distal = length of the distal phalanx of the finger Intermediate = length of the Intermediate phalanx of the finger Proximal = length of the proximal phalanx of the finger.

#### Arabs’ group

Regarding the males of the Arabs’ group in Table 3, the hand length, the total and the length of the proximal phalanx of the thumb, the total and the length of the proximal phalanx of the middle finger, the total and the length of the distal phalanx of the ring finger and the length of the proximal phalanx of the little finger showed a significant positive correlation with stature. The correlation coefficients ranged from 0.301 (for the distal phalanx of the little finger) to 0.760 (for the length of the distal phalanx of the ring finger). In the group of female Arabs, hand length, the total and the length of distal and intermediate phalanges of the index and ring fingers, all measurements of the middle finger and the total and the length of the intermediate and proximal phalanges of the little finger presented a significant positive correlation with the stature. The correlation coefficients ranged from 0.298 (for the length of the distal phalanx of the middle finger) to 0.794 (for the total length of the middle finger).

#### Malaysians’ group

Based on the results displayed in Table 3, all the studied hand measurements in the Malaysian males’ group showed a significant positive correlation with stature, except for the total and the length of the proximal phalanx of the thumb and the length of the intermediate phalanx of the little finger. The correlation coefficients ranged from 0.308 (for the length of the distal phalanx of the index finger) to 0.698 (for the total length of the index finger). In the females’ group, hand length, handbreadth, the length of the intermediate and proximal phalanges of the index finger, the total and the length of the intermediate and proximal phalanges of the middle and ring fingers and the total and the length of the proximal phalanx of the little finger revealed a significant positive correlation with the stature. The correlation coefficients ranged from 0.319 (for the length of the intermediate phalanx of the index finger) to 0.918 (for the length of the distal phalanx of the little finger).

### Simple linear regression analysis for stature estimation

Table 4 demonstrates the proposed simple linear regression equations for stature estimation in the Egyptian group in males and females separately. All the hand measurements done in the current study were used as independent variables to predict the stature.

**Table 4:** Linear regression equations for stature (S) Estimation in the egyptians’ studied group. (*n* = 100)

	Egyptians							
	Males (*n* = 50)				Females (*n* = 50)			
	Equation	*R*	*R* ^2^	*P*	Equation	*R*	*R* ^2^	*P*
*Hand L*	S = 122.89 + L*2.761	0.714	0.238	< 0.001*	S = 62.809 + L	0.669	0.447	< 0.001*
*Hand B*	S = 144.028 + B*3.718	0.273	0.075	0.055	S = 140.422 + B*2.381	0.170	0.029	0.239
*Thumb*								
Total	S = 124.881 + T*7.304	0.483	0.233	< 0.001*	S = 136.834 + T*3.557	0.268	0.072	0.060
Distal	S = 185.657 + D*1.800	0.095	0.009	0.513	S = 155.794 + D*1.257	0.082	0.007	0.573
Proximal	S = 165.986 + P*3.710	0.370	0.137	0.008*	S = 157.858 + P*0.589	0.035	0.001	0.807
*Index*								
Total	S = 173.581 + T*0.621	0.060	0.004	0.677	S = 112.204 + T*6.673	0.379	0.144	0.007*
Distal	S = 180.926 + D*0.823	0.044	0.002	0.762	S = 141.758 + D*7.467	0.386	0.149	0.006*
Intermediate	S = 184.597 + M*2.511	0.081	0.007	0.574	S = 132.386 + M*11.834	0.386	0.149	0.006*
Proximal	S = 168.127 + P*3.958	0.219	0.048	0.127	S = 164.342 + P*1.865	0.107	0.011	0.459
*Middle*								
Total	S = 143.023 + T*4.084	0.331	0.109	0.019*	S = 90.080 + T*8.995	0.794	0.631	< 0.001*
Distal	S = 187.580 + D*-3.107	0.183	0.033	0.204	S = 144.788 + D*6.056	0.298	0.089	0.035*
Intermediate	S = 168.662 + M*3.353	0.231	0.054	0.106	S = 118.524 + M*15.402	0.631	0.398	< 0.001*
Proximal	S = 160.055 + P*6.376	0.420	0.176	0.002*	S = 135.288 + P*9.489	0.562	0.316	< 0.001*
*Ring*								
Total	S = 113.070 + T*8.153	0.549	0.301	< 0.001*	S = 122.952 + T*5.386	0.430	0.185	0.002*
Distal	S = 148.077 + D*11.300	0.760	0.578	< 0.001*	S = 143.844 + D*6.275	0.340	0.116	0.016*
Intermediate	S = 195.996 + M* 5.948	0.333	0.111	0.018*	S = 110.093 + M* 21.865	0.730	0.532	< 0.001*
Proximal	S = 170.983 + P* 3.232	0.154	0.024	0.286	S = 161.391 + P* 0.776	0.058	0.003	0.687
*Little*								
Total	S = 164.538 + T* 2.103	0.186	0.034	0.197	S = 125.270 + T* 6.314	0.409	0.167	0.003*
Distal	S = 185.676 + D* 2.717	0.301	0.091	0.034*	S = 161.690 + D* 0.885	0.038	0.001	0.795
Intermediate	S = 184.190 + M* 3.003	0.123	0.015	0.393	S = 149.336 + M* 6.481	0.316	0.100	0.026*
Proximal	S = 164.426 + P* 7.104	0.534	0.285	< 0.001*	S = 150.402 + P* 5.366	0.347	0.121	0.014*

R = coefficient of correlation R^2^ = Estimate of reliability *: Statistically significant at *p* ≤ 0.05.

Hand L = hand length Hand B = hand breadth Total = total length of the finger Distal = length of the distal phalanx of the finger.

Intermediate = length of the Intermediate phalanx of the finger Proximal = length of the proximal phalanx of the finger.

In the present study, the simple linear regression equations that gave significant correlation coefficient with stature in males were the hand length with estimate of reliability (R2) = 0.238 and *p* < 0.001, the total length of the thumb and the middle fingers with R2 = 0.233 and 0.109, *p* < 0.001 and 0.019, respectively. Also, the length of the proximal phalanx of the thumb and the middle finger with R2 = 0.137 and 0.176, *p* = 0.008 and 0.002, respectively. The total length and the length of the distal and intermediate phalanges of the ring finger with R ^2^ =  0.301, 0.578, 0.111, *p* < 0.001, < 0.001 and 0.018, respectively. Moreover, the length of the distal and the proximal phalanges of the little finger with R^2^ = 0.091, 0.285, *p* = 0.034 and < 0.001.

In females, the simple linear regression equations that presented a significant estimate of reliability were hand length (R^2^ = 0.447 and *P* < 0.001). Total length, length of the distal and intermediate phalanges of the index finger (R^2^ = 0.144, 0.149 and 0.149, *p* = 0.007, 0.006 and 0.006), all the measurements of the middle finger (the total length, length of the distal, intermediate and proximal phalanges) with R^2^ = 0.631, 0.089, 0.398 and 0.316, *P* < 0.001, 0.035, < 0.001, < 0.001 respectively. For the ring finger, the total length and length of the distal and intermediate phalanges with R^2^ = 0.185, 0.116 and 0.532, *p* = 0.002, 0.016 and < 0.001, respectively. About the little finger, the total length and length of the intermediate and proximal phalanges showed a significant correlation coefficient with stature, with R^2^ = 0.167, 0.100 and 0.121, *p* = 0.003, 0.026 and 0.014.

For the Arab group, Table 5 shows the linear regression equations for stature estimation from hand measurements. In males, all the studied hand measurements were significantly correlated with stature except for the total length of the thumb, the length of the proximal phalanx of the thumb and the length of the intermediate phalanx of the little finger. The highest estimates of reliability (R2 = 0.487, 0.425, 0.414, and *p* < 0.001, < 0.001, < 0.001) were for the total length of the index, middle and ring fingers, respectively and the hand length with R2 = 0.439 and *p* < 0.001.

**Table 5:** Linear regression equations for stature (S) Estimation in the arabs’ group. (*n* = 100)

	Arabs							
	Males (*n* = 50)				Females (*n* = 50)			
	Equation	*R*	*R* ^2^	*P*	Equation	*R*	*R* ^2^	*P*
*Hand L*	S = 115.744 + L*3.028	0.663	0.439	< 0.001*	S= -4.620 + L*9.413	0.360	0.130	0.010*
*Hand B*	S = 149.845 + B*2.683	0.457	0.209	0.001*	S = 29.181 + B*24.313	0.610	0.372	< 0.001*
*Thumb*								
Total	S = 166.481 + T*1.289	0.171	0.029	0.236	S = 146.090 + T*1.333	0.036	0.001	0.804
Distal	S = 139.399 + D*9.258	0.592	0.350	< 0.001*	S = 174.310 + D*6.535	0.126	0.016	0.385
Proximal	S = 174.300 + P*0.454	0.131	0.017	0.365	S = 138.027 + P*5.528	0.137	0.019	0.343
*Index*								
Total	S = 122.239 + T*6.938	0.698	0.487	< 0.001*	S = 88.016 + T*9.963	0.247	0.061	0.083
Distal	S = 153.894 + D*7.406	0.308	0.095	0.029*	S = 301.195 + D*63.189	0.712	0.506	< 0.001*
Intermediate	S = 125.695 + M*20.363	0.655	0.429	< 0.001*	S = 104.382 + M*23.447	0.319	0.102	0.024*
Proximal	S = 163.070 + P*5.625	0.467	0.218	0.001*	S = 48.041 + P*48.557	0.690	0.476	< 0.001*
*Middle*								
Total	S = 127.089 + T*5.612	0.652	0.425	< 0.001*	S = 84.828 + T*9.535	0.337	0.113	0.017*
Distal	S = 154.490 + D*6.896	0.323	0.104	0.022*	S = 200.611 + D*20.209	0.359	0.129	0.010*
Intermediate	S = 141.263 + M*12.095	0.558	0.311	< 0.001*	S = 72.720 + M*33.185	0.647	0.419	< 0.001*
Proximal	S = 164.859 + P*4.069	0.377	0.142	0.007*	S = 80.890 + P*29.164	0.406	0.165	0.003*
*Ring*								
Total	S = 132.119 + T*5.501	0.643	0.414	< 0.001*	S = 70.657 + T*12.963	0.465	0.216	0.001*
Distal	S = 154.528 + D*7.218	0.361	0.130	0.010*	S = 216.293 + D*26.335	0.352	0.124	0.012*
Intermediate	S = 159.037 + M* 6.450	0.335	0.112	0.018*	S = 81.779 + M* 31.883	0.633	0.400	< 0.001*
Proximal	S = 166.684 + P* 3.903	0.393	0.155	0.005*	S = 36.278 + P* 65.234	0.759	0.577	< 0.001*
*Little*								
Total	S = 144.118 + T* 4.737	0.595	0.354	< 0.001*	S = 4.161 + T* 28.944	0.633	0.400	< 0.001*
Distal	S = 153.730 + D* 7.964	0.354	0.125	0.012*	S = 258.542 + D* 46.630	0.508	0.258	< 0.001*
Intermediate	S = 160.046 + M* 8.049	0.242	0.059	0.090	S = 148.268 + M* 3.581	0.045	0.002	0.757
Proximal	S = 165.299 + P* 5.451	0.531	0.282	< 0.001*	S = 95.182 + P* 44.831	0.918	0.843	< 0.001*

R: coefficient of correlation R^2^: Estimate of reliability *: Statistically significant at *p* ≤ 0.05.

Hand L = hand length Hand B = hand breadth Total = total length of the finger Distal = length of the distal phalanx of the finger.

Intermediate = length of the Intermediate phalanx of the finger Proximal = length of the proximal phalanx of the finger.

In the females, all the hand measurements showed a significant correlation with stature except for measurements of the thumb, the total length of the index finger and the length of the intermediate phalanx of the little finger. The highest correlation coefficients were for the length of the proximal phalanx of the little finger, the proximal phalanx of the ring finger, the proximal phalanx of the index finger, the intermediate phalanx of the middle finger and the handbreadth with R^2^ = 0.843, 0.577, 0.476, 0.419 and 0.372 and *p* < 0.001.

Table 6 demonstrates the linear regression equations for the Malaysian group. In males, all the studied hand measurements were significantly correlated with stature. The highest estimates of reliability were for the total length of the middle and index fingers and hand length with R2 = 0.742, 0.720, 0.703 and *p* < 0.001, respectively. In females, the highest estimates of reliability were for the intermediate phalanx of the ring finger, proximal phalanx of the thumb and little finger with R2 = 0.757, 0.695, 0.609 and *p* < 0.001, 0.000, 0.001, respectively. On the other hand, the length of the distal phalanx of the thumb and the middle fingers, the total length of the index, middle and ring fingers, the proximal phalanx of the ring finger and the intermediate phalanx of the little finger showed insignificant correlation with stature.

**Table 6:** Linear regression equations for stature (S) Estimation in the studied Malaysian group. (*n* = 100)

	Malaysians							
	Males (*n* = 50)				Females (*n* = 50)			
	Equation	*R*	*R* ^2^	*P*	Equation	*R*	*R* ^2^	*P*
*Hand L*	= 80.990 + L*4.532	0.838	0.703	< 0.001*	= 62.935 + L*5.411	0.742	0.551	< 0.001*
*Hand B*	= 138.651 + B*2.960	0.404	0.163	0.004*	= 72.561 + B*10.768	0.378	0.143	0.007*
*Thumb*								
Total	= 115.075 + T*7.881	0.673	0.453	< 0.001*	= 195.749 + T*6.647	0.652	0.425	< 0.001*
Distal	= 134.898 + D*8535	0.560	0.314	< 0.001*	= 156.602 + D*0.029	0.002	0.000	0.991
Proximal	= 151.825 + P*4.538	0.307	0.094	0.030*	= 187.385 + P*10.846	0.834	0.695	0.000*
*Index*								
Total	= 111.520 + T*7.378	0.848	0.720	< 0.001*	= 157.120 + T*0.061	0.005	0.000	0.971
Distal	= 127.463 + D*14.006	0.676	0.457	< 0.001*	= 103.265 + D*22.354	0.659	0.435	< 0.001*
Intermediate	= 142.272 + M*10.090	0.402	0.162	0.004*	= 130.980 + M*11.488	0.319	0.102	0.024*
Proximal	= 133.684 + P*13.236	0.804	0.647	< 0.001*	= 176.419 + P*8.269	0.488	0.238	< 0.001*
*Middle*								
Total	= 100.069 + T*8.082	0.861	0.742	< 0.001*	= 142.621 + T*1.748	0.186	0.035	0.196
Distal	= 131.850 + D*12.284	0.462	0.214	0.001*	= 148.363 + D*3.230	0.068	0.005	0.641
Intermediate	= 128.260 + M*13.872	0.534	0.285	< 0.001*	= 123.497 + M*12.698	0.417	0.174	0.003*
Proximal	= 131.385 + P*11.819	0.776	0.603	< 0.001*	= 152.855 + P*1.342	0.079	0.006	0.584
*Ring*								
Total	= 102.803 + T*8.264	0.775	0.600	< 0.001*	= 139.170 + T*2.508	0.197	0.039	0.170
Distal	= 135.173 + D*10.986	0.463	0.214	0.001*	= 179.604 + D*9.776	0.301	0.091	0.033*
Intermediate	= 138.490 + M* 10.568	0.495	0.245	< 0.001*	= 74.920 + M* 34.502	0.870	0.757	< 0.001*
Proximal	= 138.247 + P* 11.279	0.581	0.338	< 0.001*	= 167.695 + P* 4.727	0.175	0.031	0.224
*Little*								
Total	= 122.796 + T* 7.060	0.768	0.590	< 0.001*	= 193.499 + T* 6.719	0.468	0.219	0.001*
Distal	= 138.545 + D* 10.623	0.653	0.426	< 0.001*	= 131.962 + D* 12.254	0.350	0.123	0.013*
Intermediate	= 140.293 + M* 12.944	0.560	0.313	< 0.001*	= 158.575 + M* 1.152	0.038	0.001	0.796
Proximal	= 147.608 + P* 10.642	0.452	0.205	0.001*	= 192.716 + P* 20.539	0.780	0.609	< 0.001*

R: coefficient of correlation R^2^: Estimate of reliability *: Statistically significant at *p* ≤ 0.05.

Hand L = hand length Hand B = hand breadth Total = total length of the finger Distal = length of the distal phalanx of the finger.

Intermediate = length of the Intermediate phalanx of the finger Proximal = length of the proximal phalanx of the finger.

### Multiple regression equations for stature Estimation

Table 7 shows the multiple regression equations proposed for the estimation of stature from the studied hand measurements in the three groups. In the Egyptian and Malaysian groups, both in males and females, the multiple regression models had a value of R^2^ = one. All models were perfectly correlated with stature. In the Arabs’ group, the proposed model for the males had R2 ^2^ 0.930, while in the females’ model, the R2 = 1, with perfect correlation with stature.

**Table 7:** Multiple linear regression equations for stature Estimation developed from all the studied hand measurements, in the three studied groups.

		Equation	*R*	*R* ^2^	*P*
Egyptians	Males	29.302 + Hand.L*-30.407 + thumb.T*35.084 + thumb.P*-15.362 + Middle.T*38.319 + Middle.P*-6.544 + Ring.T*-41.309 + Ring.D*156.612 + Ring.M*102.874 + Little.D*-8.230 + Little.P*-65.019	1.000	1.000	-
	Females	-13.838 + Hand.L*14.563 + Index.T*5.653+ *-5.610 + Index.M*-2.620 + Middle.T*-65.157 + Middle.D*71.798 + Middle.M*83.098 + Middle.P*84.833 + Ring.T*-19.243 + Ring.D*3.296 + Ring.M*-29.465 + Little.T*-1.183 + Little.M*-1.008 +*-10.770	1.000	1.000	< 0.001*
Arabs	Males	52.839 + Hand.L*-4.544 + Hand.B*-4.376 + thumb.D*2.715 + Index.T*-26.518 + Index.D*57.969 + Index.M*57.869 + Index.P*47.902 + Middle.D*6.895 + Middle.M*11.001 + Middle.P*-4.075 + Ring.T*-8.485 + Ring.D*7.285 + Ring.M*13.495 + Ring.P*6.965 + Little.T*-0.985 + Little.D*-1.813 + Little.P*-5.472	0.964	0.930	< 0.001*
	Females	105.258 + Middle.M*14.871 + Middle.P*19.194 + Ring.D*-22.903 + Little.D*-8.903 + Little.P*29.097	1.000	1.000	–
Malaysians	Males	113.275 + Hand. B*0.410 + thumb. D*5.356 + Index.D*15.201 + Index.M*0.894 + Index.P*14.600 + Middle.D*-12.014 + Middle.M*-2.266 + Ring.D*1.366 + Ring.P*7.600 + Little.D*-7.100 + Little.M*1.897 + Little.P*-10.575	1.000	1.000	–
	Females	192.905 + Hand.B* -5.108 + Index.M* -2.738 + Middle.M* 9.498 + Ring.D* -15.455 + Little.D* 26.654 + Little.P* -18.605	1.000	1.000	–

R: coefficient of correlation R^2^: Coefficient of determination *: Statistically significant at *p* ≤ 0.05.

Excluded variables that were not included in the multiple linear regression equations because of collinearity and tolerance were:

In Malaysian males, hand length, total length, thumb, thumb proximal phalanx, total length, index, total length, middle, middle proximal phalanx, total length, ring, ring middle phalanx, and little total length.

In Malaysian females, hand length, total thumb length, proximal thumb length, distal index phalanx, proximal index phalanx, ring middle phalanx, and little total length.

In Arab males, the middle total length.

In Arab females, hand length, handbreadth, index distal phalanx, index middle phalanx, index proximal length, middle total length, middle distal phalanx, ring total length, ring middle phalanx, ring proximal phalanx, and little total length.

## Discussion

Stature estimation, a key aspect of forensic practice, is significantly enhanced using mathematical techniques such as regression analysis equations, which depend on hand dimensions. These equations serve as a consistent and practical tool for the estimation of stature, with direct implications for forensic investigations^31–33^.

In forensic practice, it is not uncommon to find the peripheral body parts, such as the hands or the feet, at the crime scene in cases of mass disasters and assault incidents where the body is intentionally dismembered to conceal the victim’s identity. Although geographical origin differences in anthropological studies have been noted and well-reorganised in several studies based on investigating a single population group^34–36^ comparative studies add value to the forensic identification of mass disasters in countries harbouring variant ancestries. To the best of our knowledge, limited studies were conducted on that basis^26,27,37^. The current study compares three different populations (Egyptians, Arabs from the Gulf area, and Malaysians) to postulate regression equations for predicting stature from hand dimensions, which has direct implications for forensic identification practices. Moreover, the correlation of fingers and phalanges lengths was established.

In the present study, 300 subjects, 150 males and 150 females, were recruited from Egyptian, Arab, and Malaysian medical students. Each subject’s stature and hand dimensions were measured using a manual stadiometer and a digital Vernier calliper. The choice of the right hand was based on scientific practices and comparative studies that indicated no bilateral asymmetry in hand length, unlike handbreadth, which showed asymmetry. Neglecting this asymmetry could reduce the accuracy and reliability of stature estimation, as regression formulas largely depend on the side with the strongest correlation^15,38^.

The participants in the current study were between 21 to less than 30 years old. This age group was selected to ensure the complete development, growth, and maturity of the involved subjects, as the union of epiphyses of long bones is usually completed by the age of 21 years and occurs slightly earlier in females. The upper limit for age in the selected participants was less than 30 years, a deliberate choice to avoid any potential age-related changes in stature, thereby maintaining the accuracy of the study’s findings^39^.

The current work has unveiled statistically significant differences in stature, hand length, and handbreadth among the three groups. The Egyptian group emerged as the tallest with the largest hands, followed by the Arabs and the Malaysian group. Notably, the Egyptian group exhibited the highest values in total finger length and phalanx lengths, except for the distal phalanx of the middle ring and little fingers, which were highest among the Arab group. These findings not only demonstrate ethnic disparities but also underscore the influence of physical activity, nutrition, genetics, and the environment on anthropometric measures. As a result, these traits have led to significant alterations in anthropometric dimensions, with profound implications for our understanding of human diversity and adaptation^40^.

The study by Numan et al.^41^, which compared the three major ethnic groups in Nigeria, was of significant importance. Their findings underscore the presence of discrepancies among ethnic groups, highlighting the necessity of developing equations specific to each ethnic group and both sexes.

At the same time, the present study demonstrated higher values in males than females regarding stature and all the performed hand measurements in the three studied groups. The majority were statistically significant. This result corresponded to previous studies on Egyptians^16,26,27,42^ Saudis^34^ and Malaysians^43^. In addition, Earlier observations of female hand dimensions being consistently smaller than males in different human populations were similarly reported^16,27,31,33,42,44,45^.

The present study underscores the critical necessity of developing sex-specific equations for estimating stature. This is due to the secondary influence of sex hormones, such as androgens and oestrogens, on the morphological differences between males and females. These hormones lead to an earlier onset of maturity in females and an earlier end to their growth, making sex-specific equations an urgent requirement for accurate stature estimation.

Multifaceted interactions between biology, heredity, and social and physical surroundings may help explain this. Moreover, sex hormones such as androgens and oestrogens secondarily boost the morphological discrepancy of males and females, leading to an earlier onset of maturity in females and an earlier ending of their growth than in males^46^. This highlights the pressing need for developing equations specific to sex for estimating stature^47^.

Regarding the correlation with stature, the present work has significantly contributed to understanding anthropometric variations. Only the Egyptian male group showed a positive correlation between all the hand measurements and stature, where the total length of the middle finger was followed by the total length of the index finger, and hand length demonstrated the strongest correlation with the stature. In Egyptian females, the middle phalanx of the ring finger, followed by the thumb’s proximal phalanx, the little finger’s proximal phalanx, and the hand length, showed the strongest correlation with stature. In the Arabs’ group, the distal phalanx of the ring finger and its total length, and the proximal phalanx of the little finger showed the highest correlation with stature in the males. In females, the total length of the middle finger, the middle phalanx of the ring finger, and hand length revealed the strongest correlation with stature. In the Malaysian group, the total length of the index, hand length, and total length of the ring finger in males, the proximal phalanx of the little and ring fingers, and the distal phalanx of the index in females showed the strongest correlation with the stature.

Each part of the human body develops in correlation with the other. Numerous researchers have conducted studies^19,20,29,46^ among diverse populations to find the correlation between stature and different hand measurements, consistently concluding a positive correlation. This underscores the need for further research to deepen our understanding of these relationships.

In their study, Rhiu and Kim^29^ confirmed that the finger and phalanx length significantly correlated with the stature. Gupta et al.^47^ concluded a positive and strong correlation between stature and right index finger length in females and left middle finger length in males, whereas right and left middle finger length in total subjects revealed a statistically significant correlation with stature.

Moreover, a study by Suseelamma et al.^48^ has established the left thumb length as a highly reliable indicator for stature estimation, instilling confidence in its use. Ahuja et al.^49^ further bolstered our confidence by confirming the trustworthiness of the left index and the left middle finger length in both males and females. In another study conducted in 2024, the authors concluded that the highest correlation coefficients were found for the left ring finger length in females and the left index finger length in males^50^.

At the same time, some studies were performed to demonstrate the relationship between stature and middle finger length measurement. Katwal et al.^51^ performed their study in Nepal, showing a good correlation between middle finger length and stature among males and females. A similar study was done in India^52^, where stature and middle finger length were significantly correlated, irrespective of sex. These strong correlations were utilised to develop regression equations.

On the Bangladeshi population, Asadujjaman et al.^53^ measured stature and nine hand measurements: hand length, hand breadth, maximum handbreadth, palm length, thumb length, index finger length, middle finger length, ring finger length, and little finger. They revealed that all these hand dimensions were statistically significant and positively correlated with stature. However, it’s important to note that the present study’s results contrasted with Habib and Kamal’s study^42^ on an Egyptian sample. They concluded that the measurements of the little fingers of males and those of the distal phalanges of female fingers were not correlated with stature. This discrepancy in results, possibly due to different measuring techniques, underscores the need for caution when interpreting research findings.

At the same time, in the present work, handbreadth was less correlated with stature than hand length in Egyptian males and females, and it showed no correlation with stature in the Arab group, both males and females. A similar result was obtained by Madadin and Menezes in their research^54^, in Eastern Saudi Arabia, measuring hand length, palm length, and handbreadth only, and they revealed that the hand length and palm length showed higher correlation coefficients than the handbreadth. On the other hand, this result contradicted that of Igbigbi et al.^15^ that the breadth parameters showed a stronger correlation. This variation could be due to different methodologies and different studied population groups.

In the current study, we developed simple linear regression formulae for each population for stature prediction from hand, fingers, and phalanges measurements. Our models have shown high reliability in predicting stature, providing a robust tool for forensic scientists and anthropologists. For instance, in the Egyptian group, the model of the highest estimate of reliability in males was that of the distal phalanx of the ring finger. In contrast, in Egyptian females, it was the total length of the middle finger. In the Arabs’ group, the total length of the index finger in males and the proximal phalanx of the little finger in females had the highest reliability estimate. In the Malaysian group, the total length of the middle finger in males and the middle phalanx of the ring finger in females. These results indicate that the measurements of the fingers and phalanges can be used effectively in forensic practice to predict stature. This is contrary to the results obtained by Habib and Kamal^42^ who found that hand and phalange length are reliable for estimating stature in forensic examinations, with hand length being a better predictor than phalange length.

Moreover, this study has developed multiple regression models for males and females in the three studied populations, enhancing the accuracy and precision of the models. The models of Egyptian males and females, Arab females, and Malaysian males and females provided a reliability estimate of one, indicating perfect correlation. Notably, these reliability estimates differ from those obtained by Rhiu and Kim^29^ who found that the values of the estimates of reliability derived from the multiple regression equation considering both fingers and phalanges were 0.659 for males and 0.529 for females, thereby highlighting the unique contribution of the present study.

## Conclusion

From the results of the present study, it can be concluded that the hand, even if found amputated at the crime scene, can be used to predict stature by its length, fingers, or phalanges lengths. The multiple regression models obtained from the present study were population-specific and sex-specific, and they had higher reliability estimates than the simple regression models. However, simple linear regression models can be more useful when only part of or a damaged hand is found in the crime scene, which is more common in mass disasters or murders. When the remains of a hand are found, measurements can be taken as part of the assessment. However, if the soft tissues are damaged, the assessment cannot occur on the bones, as the landmarks and dimensions are very different between intact hands and skeletonised ones. This underscores the complexity of our field and the importance of contextual information (e.g., location, clothing, personal effects) in forensic anthropology. Such information can help investigators identify the group to which the remains belong, providing a more holistic approach to our work. Our study has opened exciting avenues for future research. We recommend further investigating different age groups and comparing various population groups to enhance our understanding of this field.

## Supplementary Information

Below is the link to the electronic supplementary material.

Supplementary Material 1

## Data Availability

The authors confirm that the data supporting the findings are available from the corresponding author upon reasonable request. Data analysis is provided within the manuscript.Data is provided within the supplementary information files.
