# Supplementary material for: Stature prediction using anthropometric measurements of the hand in a sample of adult Egyptian, Arab, and Malaysian populations
Source: Sci Rep. 2025 Sep 23;15:32647. doi: 10.1038/s41598-025-15447-1 (PMC12457649; doi:10.1038/s41598-025-15447-1)
Supplement: Supplementary file 1 — Supplementary Material 1 [file 41598_2025_15447_MOESM1_ESM.pdf]

E/M	Age	Sex	Stat.	BW	Hand Measurements														
					Hand L	Hand B	thumb			Index				Middle				Ri	
							T	D	P	T	D	M	P	T	D	M	P	T	D

**M**																			
**E/M**	**Age**	**Sex**	**Stat.**	**BW**	**Hand Measurements**														
					**Hand L**	**Hand B**	**thumb**			**Index**				**Middle**				**Ri**	
							**T**	**D**	**P**	**T**	**D**	**M**	**P**	**T**	**D**	**M**	**P**	**T**	**D**
M	20	F	161	51	18.2	8	5.5	3	2.5	7.3	2.5	2.3	2.5	9.3	2.8	3	3.5	7.6	2.5
M	23	F	165	54	17.6	7.8	5.3	3	2.3	6.8	2.5	2.3	2	7.5	2.5	2.5	2.5	6.5	2
M	24	M	170.5	55	19.4	8	7	3.5	3.5	8.3	2.8	2.5	3	8.4	2.8	3	3	7.8	2.3
M	23	F	150.5	43	17	7.5	7	3.5	3.5	7.8	2.3	2.5	3	8	2.5	2.5	3	7.1	2.4

M	23	F	151	50	17.2	7.5	5.4	2.4	3	6	2	2	2	7.5	2.5	2.5	2.5	6.5	2.3
M	24	F	149	46	15.5	8	6.4	3	3.4	7.1	2.4	2	2.7	8	2.7	2.5	2.8	6.5	2.3
M	24	F	156	44	17.2	8	5.9	3.5	2.4	7	2.5	2.3	2.2	7.8	2.5	2.5	2.8	7.5	2.5
M	23	F	160	54	18.1	7.8	6	3	3	7.1	2.5	2.2	2.4	8	2.5	2.7	2.8	7.2	2.5
M	24	M	166	88	19.6	10.2	6.1	3.5	2.6	7.6	3.1	2.3	2.2	8.4	3	2.8	2.6	8.1	3
M	24	M	160	92	18	9.6	6.5	4	2.5	6.8	3	2	1.8	7.7	2.8	2.4	2.5	7.4	2.8
M	23	F	161	51	18.2	8	5.5	3	2.5	7.3	2.5	2.3	2.5	9.3	2.8	3	3.5	7.6	2.5
M	23	F	165	54	17.6	7.8	5.3	3	2.3	6.8	2.5	2.3	2	7.5	2.5	2.5	2.5	6.5	2
M	24	M	170.5	55	19.4	8	7	3.5	3.5	8.3	2.8	2.5	3	8.4	2.8	3	3	7.8	2.3
M	23	F	150.5	43	17	7.5	7	3.5	3.5	7.8	2.3	2.5	3	8	2.5	2.5	3	7.1	2.4
M	23	F	151	50	17.2	7.5	5.4	2.4	3	6	2	2	2	7.5	2.5	2.5	2.5	6.5	2.3
M	20	F	161	51	18.2	8	5.5	3	2.5	7.3	2.5	2.3	2.5	9.3	2.8	3	3.5	7.6	2.5
M	23	F	165	54	17.6	7.8	5.3	3	2.3	6.8	2.5	2.3	2	7.5	2.5	2.5	2.5	6.5	2
M	24	M	170.5	55	19.4	8	7	3.5	3.5	8.3	2.8	2.5	3	8.4	2.8	3	3	7.8	2.3
M	23	F	150.5	43	17	7.5	7	3.5	3.5	7.8	2.3	2.5	3	8	2.5	2.5	3	7.1	2.4
M	23	F	151	50	17.2	7.5	5.4	2.4	3	6	2	2	2	7.5	2.5	2.5	2.5	6.5	2.3
M	24	F	149	46	15.5	8	6.4	3	3.4	7.1	2.4	2	2.7	8	2.7	2.5	2.8	6.5	2.3
M	24	F	156	44	17.2	8	5.9	3.5	2.4	7	2.5	2.3	2.2	7.8	2.5	2.5	2.8	7.5	2.5
M	23	F	160	54	18.1	7.8	6	3	3	7.1	2.5	2.2	2.4	8	2.5	2.7	2.8	7.2	2.5
M	24	M	166	88	19.6	10.2	6.1	3.5	2.6	7.6	3.1	2.3	2.2	8.4	3	2.8	2.6	8.1	3
M	24	M	160	92	18	9.6	6.5	4	2.5	6.8	3	2	1.8	7.7	2.8	2.4	2.5	7.4	2.8
M	23	F	161	51	18.2	8	5.5	3	2.5	7.3	2.5	2.3	2.5	9.3	2.8	3	3.5	7.6	2.5
M	23	F	165	54	17.6	7.8	5.3	3	2.3	6.8	2.5	2.3	2	7.5	2.5	2.5	2.5	6.5	2
M	24	M	170.5	55	19.4	8	7	3.5	3.5	8.3	2.8	2.5	3	8.4	2.8	3	3	7.8	2.3
M	23	F	150.5	43	17	7.5	7	3.5	3.5	7.8	2.3	2.5	3	8	2.5	2.5	3	7.1	2.4
M	23	F	151	50	17.2	7.5	5.4	2.4	3	6	2	2	2	7.5	2.5	2.5	2.5	6.5	2.3
M	24	F	149	46	15.5	8	6.4	3	3.4	7.1	2.4	2	2.7	8	2.7	2.5	2.8	6.5	2.3
M	25	M	155	68	17	8	5	3	2	6	2	2	2	7	2.5	2.5	2.5	6.8	2.5
M	23	M	162	84	17.8	9.5	5.7	2.5	3.2	6.5	2.5	2	2	7.5	2.5	2.5	2.5	7.8	2.6
M	20	M	172	67	19.5	9	6.5	3.9	2.6	7.5	2.7	2.2	2.6	9	2.3	2.8	3.9	8	3
M	23	M	175	64	19.1	9	6.7	4	2.7	7.8	3	2.1	2.7	8.5	3.1	2.4	3	8	3
M	23	M	158	55	17	7.8	5.6	3.5	2.1	6.3	2.5	2	1.8	7	2.4	2.2	2.4	6	2.2
M	23	M	156	42	15.8	7.2	6	3	3	6	2	1.8	2.2	6.5	2.2	2.2	2.1	6.2	2.2

M	24	M	156	42	18	8	6.8	3.3	3.5	7.2	2.5	2.4	2.3	8	2.7	2.8	2.5	7	2.7
M	25	M	155	68	17	8	5	3	2	6	2	2	2	7	2.5	2.5	2.5	6.8	2.5
M	23	M	162	84	17.8	9.5	5.7	2.5	3.2	6.5	2.5	2	2	7.5	2.5	2.5	2.5	7.8	2.6
M	20	M	172	67	19.5	9	6.5	3.9	2.6	7.5	2.7	2.2	2.6	9	2.3	2.8	3.9	8	3
M	23	M	175	64	19.1	9	6.7	4	2.7	7.8	3	2.1	2.7	8.5	3.1	2.4	3	8	3
M	23	M	158	55	17	7.8	5.6	3.5	2.1	6.3	2.5	2	1.8	7	2.4	2.2	2.4	6	2.2
M	23	M	156	42	15.8	7.2	6	3	3	6	2	1.8	2.2	6.5	2.2	2.2	2.1	6.2	2.2
M	24	M	156	42	18	8	6.8	3.3	3.5	7.2	2.5	2.4	2.3	8	2.7	2.8	2.5	7	2.7
M	24	M	165	56.5	17.7	7.7	6.5	3.5	3	7	2.5	2	2.5	8	2.5	2.5	3	7	3
M	24	F	156	44	17.2	8	5.9	3.5	2.4	7	2.5	2.3	2.2	7.8	2.5	2.5	2.8	7.5	2.5
M	23	F	160	54	18.1	7.8	6	3	3	7.1	2.5	2.2	2.4	8	2.5	2.7	2.8	7.2	2.5
M	23	F	161	51	18.2	8	5.5	3	2.5	7.3	2.5	2.3	2.5	9.3	2.8	3	3.5	7.6	2.5
M	23	F	165	54	17.6	7.8	5.3	3	2.3	6.8	2.5	2.3	2	7.5	2.5	2.5	2.5	6.5	2
M	24	M	170.5	55	19.4	8	7	3.5	3.5	8.3	2.8	2.5	3	8.4	2.8	3	3	7.8	2.3
M	23	F	150.5	43	17	7.5	7	3.5	3.5	7.8	2.3	2.5	3	8	2.5	2.5	3	7.1	2.4
M	23	F	151	50	17.2	7.5	5.4	2.4	3	6	2	2	2	7.5	2.5	2.5	2.5	6.5	2.3
M	24	F	149	46	15.5	8	6.4	3	3.4	7.1	2.4	2	2.7	8	2.7	2.5	2.8	6.5	2.3
M	25	M	155	68	17	8	5	3	2	6	2	2	2	7	2.5	2.5	2.5	6.8	2.5
M	23	M	162	84	17.8	9.5	5.7	2.5	3.2	6.5	2.5	2	2	7.5	2.5	2.5	2.5	7.8	2.6
M	23	M	172	67	19.5	9	6.5	3.9	2.6	7.5	2.7	2.2	2.6	9	2.3	2.8	3.9	8	3
M	23	M	175	64	19.1	9	6.7	4	2.7	7.8	3	2.1	2.7	8.5	3.1	2.4	3	8	3
M	23	M	158	55	17	7.8	5.6	3.5	2.1	6.3	2.5	2	1.8	7	2.4	2.2	2.4	6	2.2
M	23	M	156	42	15.8	7.2	6	3	3	6	2	1.8	2.2	6.5	2.2	2.2	2.1	6.2	2.2
M	24	M	156	42	18	8	6.8	3.3	3.5	7.2	2.5	2.4	2.3	8	2.7	2.8	2.5	7	2.7
M	24	M	165	56.5	17.7	7.7	6.5	3.5	3	7	2.5	2	2.5	8	2.5	2.5	3	7	3
M	24	F	156	44	17.2	8	5.9	3.5	2.4	7	2.5	2.3	2.2	7.8	2.5	2.5	2.8	7.5	2.5
M	23	F	160	54	18.1	7.8	6	3	3	7.1	2.5	2.2	2.4	8	2.5	2.7	2.8	7.2	2.5
M	24	M	166	88	19.6	10.2	6.1	3.5	2.6	7.6	3.1	2.3	2.2	8.4	3	2.8	2.6	8.1	3
M	21	M	160	92	18	9.6	6.5	4	2.5	6.8	3	2	1.8	7.7	2.8	2.4	2.5	7.4	2.8
M	24	M	176	110	22	11.2	7	4	3	8.8	2.9	2.9	3	9.5	2.9	3.2	3.4	8.4	3
M	25	M	159	58	18.4	9	6.4	3.9	2.5	7.3	2.8	2.9	1.6	8.1	2.8	2.5	2.8	7.8	2.8
M	23	F	161	51	18.2	8	5.5	3	2.5	7.3	2.5	2.3	2.5	9.3	2.8	3	3.5	7.6	2.5
M	23	F	165	54	17.6	7.8	5.3	3	2.3	6.8	2.5	2.3	2	7.5	2.5	2.5	2.5	6.5	2

M	24	M	170.5	55	19.4	8	7	3.5	3.5	8.3	2.8	2.5	3	8.4	2.8	3	3	7.8	2.3
M	23	F	150.5	43	17	7.5	7	3.5	3.5	7.8	2.3	2.5	3	8	2.5	2.5	3	7.1	2.4
M	23	F	151	50	17.2	7.5	5.4	2.4	3	6	2	2	2	7.5	2.5	2.5	2.5	6.5	2.3
M	24	F	149	46	15.5	8	6.4	3	3.4	7.1	2.4	2	2.7	8	2.7	2.5	2.8	6.5	2.3
M	25	M	155	68	17	8	5	3	2	6	2	2	2	7	2.5	2.5	2.5	6.8	2.5
M	23	M	162	84	17.8	9.5	5.7	2.5	3.2	6.5	2.5	2	2	7.5	2.5	2.5	2.5	7.8	2.6
M	23	M	172	67	19.5	9	6.5	3.9	2.6	7.5	2.7	2.2	2.6	9	2.3	2.8	3.9	8	3
M	23	M	175	64	19.1	9	6.7	4	2.7	7.8	3	2.1	2.7	8.5	3.1	2.4	3	8	3
M	24	F	156	44	17.2	8	5.9	3.5	2.4	7	2.5	2.3	2.2	7.8	2.5	2.5	2.8	7.5	2.5
M	23	F	160	54	18.1	7.8	6	3	3	7.1	2.5	2.2	2.4	8	2.5	2.7	2.8	7.2	2.5
M	23	F	161	51	18.2	8	5.5	3	2.5	7.3	2.5	2.3	2.5	9.3	2.8	3	3.5	7.6	2.5
M	21	F	165	54	17.6	7.8	5.3	3	2.3	6.8	2.5	2.3	2	7.5	2.5	2.5	2.5	6.5	2
M	24	M	166	88	19.6	10.2	6.1	3.5	2.6	7.6	3.1	2.3	2.2	8.4	3	2.8	2.6	8.1	3
M	21	M	160	92	18	9.6	6.5	4	2.5	6.8	3	2	1.8	7.7	2.8	2.4	2.5	7.4	2.8
M	24	M	176	110	22	11.2	7	4	3	8.8	2.9	2.9	3	9.5	2.9	3.2	3.4	8.4	3
M	25	M	159	58	18.4	9	6.4	3.9	2.5	7.3	2.8	2.9	1.6	8.1	2.8	2.5	2.8	7.8	2.8
M	23	F	161	51	18.2	8	5.5	3	2.5	7.3	2.5	2.3	2.5	9.3	2.8	3	3.5	7.6	2.5
M	23	F	165	54	17.6	7.8	5.3	3	2.3	6.8	2.5	2.3	2	7.5	2.5	2.5	2.5	6.5	2
M	24	M	170.5	55	19.4	8	7	3.5	3.5	8.3	2.8	2.5	3	8.4	2.8	3	3	7.8	2.3
M	23	F	150.5	43	17	7.5	7	3.5	3.5	7.8	2.3	2.5	3	8	2.5	2.5	3	7.1	2.4
M	23	F	151	50	17.2	7.5	5.4	2.4	3	6	2	2	2	7.5	2.5	2.5	2.5	6.5	2.3
M	24	F	149	46	15.5	8	6.4	3	3.4	7.1	2.4	2	2.7	8	2.7	2.5	2.8	6.5	2.3
M	25	M	155	68	17	8	5	3	2	6	2	2	2	7	2.5	2.5	2.5	6.8	2.5
M	23	M	162	84	17.8	9.5	5.7	2.5	3.2	6.5	2.5	2	2	7.5	2.5	2.5	2.5	7.8	2.6
M	23	M	172	67	19.5	9	6.5	3.9	2.6	7.5	2.7	2.2	2.6	9	2.3	2.8	3.9	8	3
M	23	M	175	64	19.1	9	6.7	4	2.7	7.8	3	2.1	2.7	8.5	3.1	2.4	3	8	3
M	24	F	156	44	17.2	8	5.9	3.5	2.4	7	2.5	2.3	2.2	7.8	2.5	2.5	2.8	7.5	2.5
M	23	F	160	54	18.1	7.8	6	3	3	7.1	2.5	2.2	2.4	8	2.5	2.7	2.8	7.2	2.5
M	23	F	161	51	18.2	8	5.5	3	2.5	7.3	2.5	2.3	2.5	9.3	2.8	3	3.5	7.6	2.5
M	21	F	165	54	17.6	7.8	5.3	3	2.3	6.8	2.5	2.3	2	7.5	2.5	2.5	2.5	6.5	2
	**E**																		

E	23	F	149	40	16.8	8.5	7	3.5	3.5	7	2	2	3	7	2.3	2.5	2	6	2
E	27	F	163	82	18.5	9	7	4	3	7	2.5	2.5	2	8	3	3	2	7	3
E	23	F	166	65	18	8.5	6.8	3.8	3	7.5	3	2	3	9	3	2.6	3.4	7.5	3
E	22	F	162	68	16.5	8	6	3.2	2.8	7	2.5	2.5	2	8	2.5	2.5	3	6	2.5
E	23	F	149	40	16.8	8.5	7	3.5	3.5	7	2	2	3	7	2.3	2.5	2	6	2
E	27	F	163	82	18.5	9	7	4	3	7	2.5	2.5	2	8	3	3	2	7	3
E	23	F	166	65	18	8.5	6.8	3.8	3	7.5	3	2	3	9	3	2.6	3.4	7.5	3
E	23	F	162	68	16.5	8	6	3.2	2.8	7	2.5	2.5	2	8	2.5	2.5	3	6	2.5
E	24	M	182	85	21	9.4	7.8	3.2	4.6	7.9	2.5	2.5	3	8.3	3	2.3	3	8.3	3
E	23	M	182	78	21	9	8	4	4	8.5	3	2.5	3	9	2.2	3.8	3.5	8	3
E	22	M	169	97	18.2	9.3	7	4	3	7.4	2.8	2.2	2.4	7.9	3.1	2.5	2.3	7.4	1.9
E	26	F	163	70	19	9	6.5	3.5	3	7	2	2.5	2.5	7.5	2.5	2.5	2.5	7	2
E	23	M	184	87	19	9.3	7.5	4	3.5	7	3	2	2	8.5	3	3	2.5	8	2.5
E	26	F	158	83	17	7.8	6	3	3	7.3	3	2.1	2.3	7.5	2.6	2.4	2.5	6.7	2.5
E	26	F	158	83	17	7.8	6	3	3	7.3	3	2.1	2.3	7.5	2.6	2.4	2.5	6.7	2.5
E	22	F	166	75	18	8.5	6	2.5	3.5	7.5	2.5	2.5	2.5	8	2.5	3	2.5	7	2.5
E	27	F	166	75	18	8.5	6	2.5	3.5	7.5	2.5	2.5	2.5	8	2.5	3	2.5	7	2.5
E	25	F	159.5	73	18.5	8	7	3	4	7.5	2	2.5	3	8	2.5	3	2.5	7.5	2.5
E	26	F	146	73	16.5	7.5	6	2.5	3.5	7.5	2.5	2.5	2.5	7	2	2.5	2.5	7	2.5
E	27	F	150	65	16.5	7.8	6	3	3	6	2	2	2	6.5	2	2.5	2	6	2
E	26	F	175	65	19	7	7	2.5	3.5	7.5	2.5	2.5	2.5	8	1.5	3.5	3	7.5	2.5
E	26	F	158	83	17	7.8	6	3	3	7.3	3	2.1	2.3	7.5	2.6	2.4	2.5	6.7	2.5
E	22	F	166	75	18	8.5	6	2.5	3.5	7.5	2.5	2.5	2.5	8	2.5	3	2.5	7	2.5
E	27	F	166	75	18	8.5	6	2.5	3.5	7.5	2.5	2.5	2.5	8	2.5	3	2.5	7	2.5
E	25	F	159.5	73	18.5	8	7	3	4	7.5	2	2.5	3	8	2.5	3	2.5	7.5	2.5
E	27	F	146	73	16.5	7.5	6	2.5	3.5	7.5	2.5	2.5	2.5	7	2	2.5	2.5	7	2.5
E	27	F	150	65	16.5	7.8	6	3	3	6	2	2	2	6.5	2	2.5	2	6	2
E	27	F	175	65	19	7	7	2.5	3.5	7.5	2.5	2.5	2.5	8	1.5	3.5	3	7.5	2.5
E	23	M	174	65	21	10	7.5	4.3	3.2	8.5	3.2	2.6	2.7	9.2	3.2	3	3	8.5	2.9
E	23	M	185	80	21	9.5	7.5	4.2	3.3	8.5	3	2.5	3	9.5	3.2	3	3.3	8.7	3.2
E	23	M	178	70	19.5	9	7	3.9	3.1	8.8	3.6	2.5	2.7	8.5	2.5	3	3	8	2.5
E	23	M	173	70	19.5	9	7	4	3	7.4	2.4	2.5	2.5	9	3.4	3	2.6	8	2.5
E	23	M	170	59	20.5	8.5	7	4	3	8.3	3.2	2.6	2.5	8.7	2.7	3	3	7.6	2.4

E	23	M	174	65	21	10	7.5	4.3	3.2	8.5	3.2	2.6	2.7	9.2	3.2	3	3	8.5	2.9
E	23	M	185	80	21	9.5	7.5	4.2	3.3	8.5	3	2.5	3	9.5	3.2	3	3.3	8.7	3.2
E	23	M	178	70	19.5	9	7	3.9	3.1	8.8	3.6	2.5	2.7	8.5	2.5	3	3	8	2.5
E	23	M	173	70	19.5	9	7	4	3	7.4	2.4	2.5	2.5	9	3.4	3	2.6	8	2.5
E	23	M	170	59	20.5	8.5	7	4	3	8.3	3.2	2.6	2.5	8.7	2.7	3	3	7.6	2.4
E	24	F	162	71	18.5	8.3	6.2	3.2	3	7.2	2.4	2.3	2.5	7.6	2.5	2.5	2.6	7	2.5
E	24	M	184	99	21	10.3	6.9	4.3	2.6	8	2.8	2.5	2.7	9.3	3.1	2.9	3.3	8.5	3.1
E	23	M	184	75	21	9	6.9	4.2	2.7	7.7	2.8	2.5	2.4	8.3	2.9	2.9	2.5	7.5	3
E	22	F	154	60	17.4	7.6	5.5	3.1	2.4	6.6	2	2.2	2.4	7.5	2.4	2.5	2.6	7.6	3.6
E	22	F	160	58	17.8	7.7	6.5	3.2	3.3	7.4	2	2.5	2.9	7.7	2.4	2.5	2.8	7	2.3
E	23	F	162	68	16.5	8	6	3.2	2.8	7	2.5	2.5	2	8	2.5	2.5	3	6	2.5
E	24	F	166	92	17.7	8	7.5	3.5	4	6.8	2.6	2.5	1.7	8.7	2.7	3	3	7	2.9
E	23	F	160	70	18	8	7	3	4	6.8	2	2	2.8	7.5	2.5	2.5	2.5	7.3	2.4
E	23	F	149	40	16.8	8.5	7	3.5	3.5	7	2	2	3	7	2.3	2.5	2	6	2
E	24	F	163	82	18.5	9	7	4	3	7	2.5	2.5	2	8	3	3	2	7	3
E	23	F	166	65	18	8.5	6.8	3.8	3	7.5	3	2	3	9	3	2.6	3.4	7.5	3
E	23	F	162	68	16.5	8	6	3.2	2.8	7	2.5	2.5	2	8	2.5	2.5	3	6	2.5
E	22	F	154	60	17.4	7.6	5.5	3.1	2.4	6.6	2	2.2	2.4	7.5	2.4	2.5	2.6	7.6	3.6
E	22	F	160	58	17.8	7.7	6.5	3.2	3.3	7.4	2	2.5	2.9	7.7	2.4	2.5	2.8	7	2.3
E	23	F	162	68	16.5	8	6	3.2	2.8	7	2.5	2.5	2	8	2.5	2.5	3	6	2.5
E	24	F	166	92	17.7	8	7.5	3.5	4	6.8	2.6	2.5	1.7	8.7	2.7	3	3	7	2.9
E	23	F	160	70	18	8	7	3	4	6.8	2	2	2.8	7.5	2.5	2.5	2.5	7.3	2.4
E	23	F	149	40	16.8	8.5	7	3.5	3.5	7	2	2	3	7	2.3	2.5	2	6	2
E	24	F	163	82	18.5	9	7	4	3	7	2.5	2.5	2	8	3	3	2	7	3
E	23	F	166	65	18	8.5	6.8	3.8	3	7.5	3	2	3	9	3	2.6	3.4	7.5	3
E	23	F	162	68	16.5	8	6	3.2	2.8	7	2.5	2.5	2	8	2.5	2.5	3	6	2.5
E	23	M	182	85	21	9.4	7.8	3.2	4.6	7.9	2.5	2.5	3	8.3	3	2.3	3	8.3	3
E	23	M	182	78	21	9	8	4	4	8.5	3	2.5	3	9	2.2	3.8	3.5	8	3
E	22	M	169	97	18.2	9.3	7	4	3	7.4	2.8	2.2	2.4	7.9	3.1	2.5	2.3	7.4	1.9
E	25	F	163	70	19	9	6.5	3.5	3	7	2	2.5	2.5	7.5	2.5	2.5	2.5	7	2
E	23	M	184	87	19	9.3	7.5	4	3.5	7	3	2	2	8.5	3	3	2.5	8	2.5
E	23	F	158	83	17	7.8	6	3	3	7.3	3	2.1	2.3	7.5	2.6	2.4	2.5	6.7	2.5
E	22	F	158	83	17	7.8	6	3	3	7.3	3	2.1	2.3	7.5	2.6	2.4	2.5	6.7	2.5

E	23	F	166	75	18	8.5	6	2.5	3.5	7.5	2.5	2.5	2.5	8	2.5	3	2.5	7	2.5
E	20	F	159.5	73	18.5	8	7	3	4	7.5	2	2.5	3	8	2.5	3	2.5	7.5	2.5
E	27	F	146	73	16.5	7.5	6	2.5	3.5	7.5	2.5	2.5	2.5	7	2	2.5	2.5	7	2.5
E	27	F	150	65	16.5	7.8	6	3	3	6	2	2	2	6.5	2	2.5	2	6	2
E	23	M	182	85	21	9.4	7.8	3.2	4.6	7.9	2.5	2.5	3	8.3	3	2.3	3	8.3	3
E	23	M	182	78	21	9	8	4	4	8.5	3	2.5	3	9	2.2	3.8	3.5	8	3
E	22	M	169	97	18.2	9.3	7	4	3	7.4	2.8	2.2	2.4	7.9	3.1	2.5	2.3	7.4	1.9
E	23	M	184	87	19	9.3	7.5	4	3.5	7	3	2	2	8.5	3	3	2.5	8	2.5
E	23	M	174	65	21	10	7.5	4.3	3.2	8.5	3.2	2.6	2.7	9.2	3.2	3	3	8.5	2.9
E	23	M	185	80	21	9.5	7.5	4.2	3.3	8.5	3	2.5	3	9.5	3.2	3	3.3	8.7	3.2
E	23	M	178	70	19.5	9	7	3.9	3.1	8.8	3.6	2.5	2.7	8.5	2.5	3	3	8	2.5
E	23	M	173	70	19.5	9	7	4	3	7.4	2.4	2.5	2.5	9	3.4	3	2.6	8	2.5
E	23	M	170	59	20.5	8.5	7	4	3	8.3	3.2	2.6	2.5	8.7	2.7	3	3	7.6	2.4
E	24	M	184	99	21	10.3	6.9	4.3	2.6	8	2.8	2.5	2.7	9.3	3.1	2.9	3.3	8.5	3.1
E	23	M	184	75	21	9	6.9	4.2	2.7	7.7	2.8	2.5	2.4	8.3	2.9	2.9	2.5	7.5	3
E	23	M	182	85	21	9.4	7.8	3.2	4.6	7.9	2.5	2.5	3	8.3	3	2.3	3	8.3	3
E	23	M	182	78	21	9	8	4	4	8.5	3	2.5	3	9	2.2	3.8	3.5	8	3
E	22	M	169	97	18.2	9.3	7	4	3	7.4	2.8	2.2	2.4	7.9	3.1	2.5	2.3	7.4	1.9
E	23	M	184	87	19	9.3	7.5	4	3.5	7	3	2	2	8.5	3	3	2.5	8	2.5
E	23	M	182	85	21	9.4	7.8	3.2	4.6	7.9	2.5	2.5	3	8.3	3	2.3	3	8.3	3
E	23	M	182	78	21	9	8	4	4	8.5	3	2.5	3	9	2.2	3.8	3.5	8	3
E	22	M	169	97	18.2	9.3	7	4	3	7.4	2.8	2.2	2.4	7.9	3.1	2.5	2.3	7.4	1.9
E	23	M	184	87	19	9.3	7.5	4	3.5	7	3	2	2	8.5	3	3	2.5	8	2.5
E	23	M	174	65	21	10	7.5	4.3	3.2	8.5	3.2	2.6	2.7	9.2	3.2	3	3	8.5	2.9
E	23	M	185	80	21	9.5	7.5	4.2	3.3	8.5	3	2.5	3	9.5	3.2	3	3.3	8.7	3.2
E	23	M	178	70	19.5	9	7	3.9	3.1	8.8	3.6	2.5	2.7	8.5	2.5	3	3	8	2.5
E	23	M	173	70	19.5	9	7	4	3	7.4	2.4	2.5	2.5	9	3.4	3	2.6	8	2.5
E	23	M	170	59	20.5	8.5	7	4	3	8.3	3.2	2.6	2.5	8.7	2.7	3	3	7.6	2.4
E	24	M	184	99	21	10.3	6.9	4.3	2.6	8	2.8	2.5	2.7	9.3	3.1	2.9	3.3	8.5	3.1
E	23	M	184	75	21	9	6.9	4.2	2.7	7.7	2.8	2.5	2.4	8.3	2.9	2.9	2.5	7.5	3
E	23	M	182	85	21	9.4	7.8	3.2	4.6	7.9	2.5	2.5	3	8.3	3	2.3	3	8.3	3
E	23	M	182	78	21	9	8	4	4	8.5	3	2.5	3	9	2.2	3.8	3.5	8	3
E	22	M	169	97	18.2	9.3	7	4	3	7.4	2.8	2.2	2.4	7.9	3.1	2.5	2.3	7.4	1.9

E	23	M	184	87	19	9.3	7.5	4	3.5	7	3	2	2	8.5	3	3	2.5	8	2.5

A

A	23	M	168	70	19	9.8	7	3.5	3.5	7.9	3	2.5	2.1	8.8	3	2.9	2.9	8	3
A	27	M	173	62	19.5	9.5	6.5	4	2.5	7.5	3	2.4	2.1	8.5	3	2.8	2.7	7.8	3
A	23	M	168	92	18.7	10	6.5	3.7	2.8	7	2.5	2.4	2.1	8.2	2.8	2.9	2.5	7.5	2.8
A	25	M	166	63	19	9	7	4	3	7.5	2.5	2.5	2.5	8	2.5	2.5	3	7.2	2.7
A	23	M	172	85	18.8	8.8	7.2	3.5	3.7	7	3	2.4	1.6	7.5	3	2.3	2.2	6.7	3
A	23	F	147	50	16	7	6	3.5	2.5	6.5	2.3	2	2.2	7	2.2	2.2	2.6	6	2.2
A	23	F	135.5	53	17	7.5	6	3	3	6.5	2.5	2	2	7	2.5	2.2	2.3	6.2	2.5
A	23	M	171	76	20	9.2	7	4	3	8	3	2.3	2.7	9	3	2.6	3.4	8.1	3
A	22	M	174	90	19.2	9	7	3.5	3.5	7.2	3	2.5	1.7	8.2	3	3	2.2	7.7	3
A	22	M	173	68	19.1	10	7	3.5	3.5	7.3	2.8	2.3	2.2	8	3	2.9	2.1	7.6	2.7
A	24	M	180	64	22	10	7.8	4	3.8	8.5	3	2.6	2.9	9.5	3	3.1	3.4	9	3
A	23	M	178	85	20	9	7.3	4	3.3	7.8	3.1	2.5	2.2	9	3.3	2.9	2.8	8.3	3.1
A	23	M	172	74	20	9	8.8	4	4.8	7.5	3.2	2.4	1.9	8.5	3.5	2.5	2.5	7.6	3.1
A	24	M	176	84	20	10.5	7	4	3	8	2.9	2.5	2.6	9	3.2	3	2.8	8.1	3
A	22	M	180	105	20.5	10.5	8	4	8	8.1	3.1	2.5	2.5	8.8	3.4	2.9	2.5	8.2	3.2
A	22	M	175	85	19.5	9.7	7.5	4	3.5	7.5	3	2.2	2.3	8.5	3.1	2.9	2.5	7.9	2.5
A	26	M	185	120	21	10.6	7.4	4.5	2.9	8.3	3	2.8	2.5	9.5	3.2	3	3.3	8.5	3
A	23	M	179	135	19.5	11	7.5	4	3.5	8.1	3	2.6	2.5	9	3	3	3	8	2.8
A	23	M	177	98	20.5	10.5	7	4.5	2.5	7.9	3.3	2.5	2.1	9	3.2	2.9	2.9	8.5	3.4
A	23	M	171	76	20	9.2	7	4	3	8	3	2.3	2.7	9	3	2.6	3.4	8.1	3
A	22	M	174	90	19.2	9	7	3.5	3.5	7.2	3	2.5	1.7	8.2	3	3	2.2	7.7	3
A	22	M	173	68	19.1	10	7	3.5	3.5	7.3	2.8	2.3	2.2	8	3	2.9	2.1	7.6	2.7
A	24	M	180	64	22	10	7.8	4	3.8	8.5	3	2.6	2.9	9.5	3	3.1	3.4	9	3
A	23	M	178	85	20	9	7.3	4	3.3	7.8	3.1	2.5	2.2	9	3.3	2.9	2.8	8.3	3.1
A	23	M	172	74	20	9	8.8	4	4.8	7.5	3.2	2.4	1.9	8.5	3.5	2.5	2.5	7.6	3.1
A	24	M	176	84	20	10.5	7	4	3	8	2.9	2.5	2.6	9	3.2	3	2.8	8.1	3
A	22	M	180	105	20.5	10.5	8	4	8	8.1	3.1	2.5	2.5	8.8	3.4	2.9	2.5	8.2	3.2
A	22	M	175	85	19.5	9.7	7.5	4	3.5	7.5	3	2.2	2.3	8.5	3.1	2.9	2.5	7.9	2.5
A	26	M	185	120	21	10.6	7.4	4.5	2.9	8.3	3	2.8	2.5	9.5	3.2	3	3.3	8.5	3

A	23	M	179	135	19.5	11	7.5	4	3.5	8.1	3	2.6	2.5	9	3	3	3	8	2.8
A	23	M	177	98	20.5	10.5	7	4.5	2.5	7.9	3.3	2.5	2.1	9	3.2	2.9	2.9	8.5	3.4
A	27	F	185	53	17	8	6	3.2	2.8	6.5	2	2	2.5	7	2	2.5	2.5	6.5	2
A	22	M	185.5	84	21.3	9.2	7.2	4.2	3	8.2	3	2.5	2.7	8.5	3.2	3	2.3	8	3.6
A	23	F	167	78	18	8	6.5	3	3.5	7.5	2.4	2.5	2.6	8.7	2.7	3	3	7.7	2.7
A	22	F	161	55	17	7.4	5	2.5	2.5	6.1	2.1	2	2	7	1.8	2.9	2.3	6	2.3
A	23	F	159	55	17	8	6.5	3	3.5	7	2.5	2.5	2	7.5	2.5	2.5	2.5	7	2.5
A	26	M	175	75	18	8.2	7	4	3	7.3	2.5	2.3	2.5	8	2.8	2.5	2.7	7	2.8
A	25	M	175	78	19	9.5	6.5	4	2.5	7.2	3	2.5	1.7	8.8	3	3	2.8	7.5	2.9
A	22	M	174	90	19.2	9	7	3.5	3.5	7.2	3	2.5	1.7	8.2	3	3	2.2	7.7	3
A	22	M	173	68	19.1	10	7	3.5	3.5	7.3	2.8	2.3	2.2	8	3	2.9	2.1	7.6	2.7
A	24	M	180	64	22	10	7.8	4	3.8	8.5	3	2.6	2.9	9.5	3	3.1	3.4	9	3
A	23	M	178	85	20	9	7.3	4	3.3	7.8	3.1	2.5	2.2	9	3.3	2.9	2.8	8.3	3.1
A	23	M	172	74	20	9	8.8	4	4.8	7.5	3.2	2.4	1.9	8.5	3.5	2.5	2.5	7.6	3.1
A	24	M	176	84	20	10.5	7	4	3	8	2.9	2.5	2.6	9	3.2	3	2.8	8.1	3
A	26	M	175	75	18	8.2	7	4	3	7.3	2.5	2.3	2.5	8	2.8	2.5	2.7	7	2.8
A	25	M	175	78	19	9.5	6.5	4	2.5	7.2	3	2.5	1.7	8.8	3	3	2.8	7.5	2.9
A	22	M	174	90	19.2	9	7	3.5	3.5	7.2	3	2.5	1.7	8.2	3	3	2.2	7.7	3
A	22	M	173	68	19.1	10	7	3.5	3.5	7.3	2.8	2.3	2.2	8	3	2.9	2.1	7.6	2.7
A	24	M	180	64	22	10	7.8	4	3.8	8.5	3	2.6	2.9	9.5	3	3.1	3.4	9	3
A	23	M	178	85	20	9	7.3	4	3.3	7.8	3.1	2.5	2.2	9	3.3	2.9	2.8	8.3	3.1
A	23	M	172	74	20	9	8.8	4	4.8	7.5	3.2	2.4	1.9	8.5	3.5	2.5	2.5	7.6	3.1
A	24	M	176	84	20	10.5	7	4	3	8	2.9	2.5	2.6	9	3.2	3	2.8	8.1	3
A	22	M	180	105	20.5	10.5	8	4	8	8.1	3.1	2.5	2.5	8.8	3.4	2.9	2.5	8.2	3.2
A	22	M	175	85	19.5	9.7	7.5	4	3.5	7.5	3	2.2	2.3	8.5	3.1	2.9	2.5	7.9	2.5
A	26	M	185	120	21	10.6	7.4	4.5	2.9	8.3	3	2.8	2.5	9.5	3.2	3	3.3	8.5	3
A	23	M	179	135	19.5	11	7.5	4	3.5	8.1	3	2.6	2.5	9	3	3	3	8	2.8
A	27	F	185	53	17	8	6	3.2	2.8	6.5	2	2	2.5	7	2	2.5	2.5	6.5	2
A	23	F	167	78	18	8	6.5	3	3.5	7.5	2.4	2.5	2.6	8.7	2.7	3	3	7.7	2.7
A	22	F	161	55	17	7.4	5	2.5	2.5	6.1	2.1	2	2	7	1.8	2.9	2.3	6	2.3
A	23	F	159	55	17	8	6.5	3	3.5	7	2.5	2.5	2	7.5	2.5	2.5	2.5	7	2.5
A	27	F	185	53	17	8	6	3.2	2.8	6.5	2	2	2.5	7	2	2.5	2.5	6.5	2
A	23	F	167	78	18	8	6.5	3	3.5	7.5	2.4	2.5	2.6	8.7	2.7	3	3	7.7	2.7

A	22	F	161	55	17	7.4	5	2.5	2.5	6.1	2.1	2	2	7	1.8	2.9	2.3	6	2.3
A	23	F	159	55	17	8	6.5	3	3.5	7	2.5	2.5	2	7.5	2.5	2.5	2.5	7	2.5
A	27	F	185	53	17	8	6	3.2	2.8	6.5	2	2	2.5	7	2	2.5	2.5	6.5	2
A	23	F	167	78	18	8	6.5	3	3.5	7.5	2.4	2.5	2.6	8.7	2.7	3	3	7.7	2.7
A	22	F	161	55	17	7.4	5	2.5	2.5	6.1	2.1	2	2	7	1.8	2.9	2.3	6	2.3
A	23	F	159	55	17	8	6.5	3	3.5	7	2.5	2.5	2	7.5	2.5	2.5	2.5	7	2.5
A	23	F	147	50	16	7	6	3.5	2.5	6.5	2.3	2	2.2	7	2.2	2.2	2.6	6	2.2
A	23	F	135.5	53	17	7.5	6	3	3	6.5	2.5	2	2	7	2.5	2.2	2.3	6.2	2.5
A	23	F	147	50	16	7	6	3.5	2.5	6.5	2.3	2	2.2	7	2.2	2.2	2.6	6	2.2
A	23	F	135.5	53	17	7.5	6	3	3	6.5	2.5	2	2	7	2.5	2.2	2.3	6.2	2.5
A	23	F	147	50	16	7	6	3.5	2.5	6.5	2.3	2	2.2	7	2.2	2.2	2.6	6	2.2
A	23	F	135.5	53	17	7.5	6	3	3	6.5	2.5	2	2	7	2.5	2.2	2.3	6.2	2.5
A	23	F	147	50	16	7	6	3.5	2.5	6.5	2.3	2	2.2	7	2.2	2.2	2.6	6	2.2
A	23	F	135.5	53	17	7.5	6	3	3	6.5	2.5	2	2	7	2.5	2.2	2.3	6.2	2.5
A	23	F	147	50	16	7	6	3.5	2.5	6.5	2.3	2	2.2	7	2.2	2.2	2.6	6	2.2
A	23	F	135.5	53	17	7.5	6	3	3	6.5	2.5	2	2	7	2.5	2.2	2.3	6.2	2.5
A	23	F	147	50	16	7	6	3.5	2.5	6.5	2.3	2	2.2	7	2.2	2.2	2.6	6	2.2
A	23	F	135.5	53	17	7.5	6	3	3	6.5	2.5	2	2	7	2.5	2.2	2.3	6.2	2.5
A	27	F	185	53	17	8	6	3.2	2.8	6.5	2	2	2.5	7	2	2.5	2.5	6.5	2
A	23	F	167	78	18	8	6.5	3	3.5	7.5	2.4	2.5	2.6	8.7	2.7	3	3	7.7	2.7
A	22	F	161	55	17	7.4	5	2.5	2.5	6.1	2.1	2	2	7	1.8	2.9	2.3	6	2.3
A	23	F	159	55	17	8	6.5	3	3.5	7	2.5	2.5	2	7.5	2.5	2.5	2.5	7	2.5
A	27	F	185	53	17	8	6	3.2	2.8	6.5	2	2	2.5	7	2	2.5	2.5	6.5	2
A	23	F	167	78	18	8	6.5	3	3.5	7.5	2.4	2.5	2.6	8.7	2.7	3	3	7.7	2.7
A	22	F	161	55	17	7.4	5	2.5	2.5	6.1	2.1	2	2	7	1.8	2.9	2.3	6	2.3
A	23	F	159	55	17	8	6.5	3	3.5	7	2.5	2.5	2	7.5	2.5	2.5	2.5	7	2.5
A	23	F	147	50	16	7	6	3.5	2.5	6.5	2.3	2	2.2	7	2.2	2.2	2.6	6	2.2
A	23	F	135.5	53	17	7.5	6	3	3	6.5	2.5	2	2	7	2.5	2.2	2.3	6.2	2.5
A	23	F	147	50	16	7	6	3.5	2.5	6.5	2.3	2	2.2	7	2.2	2.2	2.6	6	2.2
A	23	F	135.5	53	17	7.5	6	3	3	6.5	2.5	2	2	7	2.5	2.2	2.3	6.2	2.5
A	23	F	147	50	16	7	6	3.5	2.5	6.5	2.3	2	2.2	7	2.2	2.2	2.6	6	2.2
A	23	F	135.5	53	17	7.5	6	3	3	6.5	2.5	2	2	7	2.5	2.2	2.3	6.2	2.5
A	23	F	147	50	16	7	6	3.5	2.5	6.5	2.3	2	2.2	7	2.2	2.2	2.6	6	2.2

A	23	F	135.5	53	17	7.5	6	3	3	6.5	2.5	2	2	7	2.5	2.2	2.3	6.2	2.5
A	23	F	147	50	16	7	6	3.5	2.5	6.5	2.3	2	2.2	7	2.2	2.2	2.6	6	2.2
A	23	F	135.5	53	17	7.5	6	3	3	6.5	2.5	2	2	7	2.5	2.2	2.3	6.2	2.5
A	23	F	147	50	16	7	6	3.5	2.5	6.5	2.3	2	2.2	7	2.2	2.2	2.6	6	2.2
A	23	F	135.5	53	17	7.5	6	3	3	6.5	2.5	2	2	7	2.5	2.2	2.3	6.2	2.5

ng		Little			
M	P	T	D	M	P

ng		Little			
M	P	T	D	M	P
2.5	2.6	5	2	1.5	1.5
2.5	2	5	2	1.5	1.5
3	2.5	6.5	2.5	2	2
2.2	2.5	5.5	2	1.5	2

2.3	2.3	5.6	1.7	1.7	1.7
2.1	2.1	5.6	2	1.5	2.1
2.5	2.5	6	2.2	2	1.8
2.4	2.3	6.1	2.3	1.9	1.9
2.6	2.5	7	2.7	2.6	1.7
2.1	2.5	6	2.5	1.5	2
2.5	2.6	5	2	1.5	1.5
2.5	2	5	2	1.5	1.5
3	2.5	6.5	2.5	2	2
2.2	2.5	5.5	2	1.5	2
2.3	2.3	5.6	1.7	1.7	1.7
2.5	2.6	5	2	1.5	1.5
2.5	2	5	2	1.5	1.5
3	2.5	6.5	2.5	2	2
2.2	2.5	5.5	2	1.5	2
2.3	2.3	5.6	1.7	1.7	1.7
2.1	2.1	5.6	2	1.5	2.1
2.5	2.5	6	2.2	2	1.8
2.4	2.3	6.1	2.3	1.9	1.9
2.6	2.5	7	2.7	2.6	1.7
2.1	2.5	6	2.5	1.5	2
2.5	2.6	5	2	1.5	1.5
2.5	2	5	2	1.5	1.5
3	2.5	6.5	2.5	2	2
2.2	2.5	5.5	2	1.5	2
2.3	2.3	5.6	1.7	1.7	1.7
2.1	2.1	5.6	2	1.5	2.1
2.3	2	4.5	2	1.5	1
2.5	2.7	5.1	1.6	1.8	1.7
2.7	2.3	6.6	3	2	1.6
2.1	2.9	6.3	3	1.8	1.5
2	1.8	5.3	2.5	1.7	1.1
2	2	5	2	1.5	1.5

2.5	1.8	5.8	2.4	1.8	1.6
2.3	2	4.5	2	1.5	1
2.5	2.7	5.1	1.6	1.8	1.7
2.7	2.3	6.6	3	2	1.6
2.1	2.9	6.3	3	1.8	1.5
2	1.8	5.3	2.5	1.7	1.1
2	2	5	2	1.5	1.5
2.5	1.8	5.8	2.4	1.8	1.6
2.5	1.5	5.5	2	2	1.5
2.5	2.5	6	2.2	2	1.8
2.4	2.3	6.1	2.3	1.9	1.9
2.5	2.6	5	2	1.5	1.5
2.5	2	5	2	1.5	1.5
3	2.5	6.5	2.5	2	2
2.2	2.5	5.5	2	1.5	2
2.3	2.3	5.6	1.7	1.7	1.7
2.1	2.1	5.6	2	1.5	2.1
2.3	2	4.5	2	1.5	1
2.5	2.7	5.1	1.6	1.8	1.7
2.7	2.3	6.6	3	2	1.6
2.1	2.9	6.3	3	1.8	1.5
2	1.8	5.3	2.5	1.7	1.1
2	2	5	2	1.5	1.5
2.5	1.8	5.8	2.4	1.8	1.6
2.5	1.5	5.5	2	2	1.5
2.5	2.5	6	2.2	2	1.8
2.4	2.3	6.1	2.3	1.9	1.9
2.6	2.5	7	2.7	2.6	1.7
2.1	2.5	6	2.5	1.5	2
3	2.4	7	2.9	2.4	1.7
2.5	2.5	6.2	2.8	2	1.4
2.5	2.6	5	2	1.5	1.5
2.5	2	5	2	1.5	1.5

3	2.5	6.5	2.5	2	2
2.2	2.5	5.5	2	1.5	2
2.3	2.3	5.6	1.7	1.7	1.7
2.1	2.1	5.6	2	1.5	2.1
2.3	2	4.5	2	1.5	1
2.5	2.7	5.1	1.6	1.8	1.7
2.7	2.3	6.6	3	2	1.6
2.1	2.9	6.3	3	1.8	1.5
2.5	2.5	6	2.2	2	1.8
2.4	2.3	6.1	2.3	1.9	1.9
2.5	2.6	5	2	1.5	1.5
2.5	2	5	2	1.5	1.5
2.6	2.5	7	2.7	2.6	1.7
2.1	2.5	6	2.5	1.5	2
3	2.4	7	2.9	2.4	1.7
2.5	2.5	6.2	2.8	2	1.4
2.5	2.6	5	2	1.5	1.5
2.5	2	5	2	1.5	1.5
3	2.5	6.5	2.5	2	2
2.2	2.5	5.5	2	1.5	2
2.3	2.3	5.6	1.7	1.7	1.7
2.1	2.1	5.6	2	1.5	2.1
2.3	2	4.5	2	1.5	1
2.5	2.7	5.1	1.6	1.8	1.7
2.7	2.3	6.6	3	2	1.6
2.1	2.9	6.3	3	1.8	1.5
2.5	2.5	6	2.2	2	1.8
2.4	2.3	6.1	2.3	1.9	1.9
2.5	2.6	5	2	1.5	1.5
2.5	2	5	2	1.5	1.5

2	2	5	2	1.5	1.5
2	2	6	1.5	1.5	3
2.4	2.5	5.5	2.5	1.8	2.1
2.5	1	5	2.5	1	1.5
2	2	5	2	1.5	1.5
2	2	6	1.5	1.5	3
2.4	2.5	5.5	2.5	1.8	2.1
2.5	1	5	2.5	1	1.5
3	2.1	6.5	2.7	2.2	1.9
2.5	2.5	7	2	1.5	2
3.3	2.2	6	2.6	1.8	1.6
2.5	2.5	6	2	2	2
3.5	2	6	1.5	1.5	3
2.1	2.1	5.2	2.3	1.5	1.3
2.1	2.1	5.2	2.3	1.5	1.3
2.5	2	5	2	1.5	1.5
2.5	2	5	2	1.5	1.5
2.5	3	6	2	2	2
2	2.5	5.5	2.5	1.5	1.5
2	2	5	2	1.5	1.5
2.5	2.5	6.5	2	2.5	2
2.1	2.1	5.2	2.3	1.5	1.3
2.5	2	5	2	1.5	1.5
2.5	2	5	2	1.5	1.5
2.5	3	6	2	2	2
2	2.5	5.5	2.5	1.5	1.5
2	2	5	2	1.5	1.5
2.5	2.5	6.5	2	2.5	2
2.8	2.3	7	3	2	2
2.5	3	7	2.8	2.1	2.1
3	2.5	7.7	4.2	2	1.5
3	2.5	7	2.9	2	2.1
3	2.2	6.2	2.7	2	1.5

2.8	2.3	7	3	2	2
2.5	3	7	2.8	2.1	2.1
3	2.5	7.7	4.2	2	1.5
3	2.5	7	2.9	2	2.1
3	2.2	6.2	2.7	2	1.5
2.5	2	5.7	2.1	1.9	1.7
2.8	2.6	7	2.8	2	2.2
2.5	2	6.3	2.7	2	1.6
2	2	5.5	2.1	1.9	1.5
2.3	2.4	5.9	2.1	1.8	2
2.5	1	5	2.5	1	1.5
2.6	1.5	6	2.6	2	1.4
2.1	2.8	5.6	2.4	1.5	1.7
2	2	5	2	1.5	1.5
2	2	6	1.5	1.5	3
2.4	2.5	5.5	2.5	1.8	2.1
2.5	1	5	2.5	1	1.5
2	2	5.5	2.1	1.9	1.5
2.3	2.4	5.9	2.1	1.8	2
2.5	1	5	2.5	1	1.5
2.6	1.5	6	2.6	2	1.4
2.1	2.8	5.6	2.4	1.5	1.7
2	2	5	2	1.5	1.5
2	2	6	1.5	1.5	3
2.4	2.5	5.5	2.5	1.8	2.1
2.5	1	5	2.5	1	1.5
3	2.1	6.5	2.7	2.2	1.9
2.5	2.5	7	2	1.5	2
3.3	2.2	6	2.6	1.8	1.6
2.5	2.5	6	2	2	2
3.5	2	6	1.5	1.5	3
2.1	2.1	5.2	2.3	1.5	1.3
2.1	2.1	5.2	2.3	1.5	1.3

2.5	2	5	2	1.5	1.5
2.5	3	6	2	2	2
2	2.5	5.5	2.5	1.5	1.5
2	2	5	2	1.5	1.5
3	2.1	6.5	2.7	2.2	1.9
2.5	2.5	7	2	1.5	2
3.3	2.2	6	2.6	1.8	1.6
3.5	2	6	1.5	1.5	3
2.8	2.3	7	3	2	2
2.5	3	7	2.8	2.1	2.1
3	2.5	7.7	4.2	2	1.5
3	2.5	7	2.9	2	2.1
3	2.2	6.2	2.7	2	1.5
2.8	2.6	7	2.8	2	2.2
2.5	2	6.3	2.7	2	1.6
3	2.1	6.5	2.7	2.2	1.9
2.5	2.5	7	2	1.5	2
3.3	2.2	6	2.6	1.8	1.6
3.5	2	6	1.5	1.5	3
3	2.1	6.5	2.7	2.2	1.9
2.5	2.5	7	2	1.5	2
3.3	2.2	6	2.6	1.8	1.6
3.5	2	6	1.5	1.5	3
2.8	2.3	7	3	2	2
2.5	3	7	2.8	2.1	2.1
3	2.5	7.7	4.2	2	1.5
3	2.5	7	2.9	2	2.1
3	2.2	6.2	2.7	2	1.5
2.8	2.6	7	2.8	2	2.2
2.5	2	6.3	2.7	2	1.6
3	2.1	6.5	2.7	2.2	1.9
2.5	2.5	7	2	1.5	2
3.3	2.2	6	2.6	1.8	1.6

3.5	2	6	1.5	1.5	3

2.5	2.5	6.6	3	2	1.6
2.6	2.2	6.5	2.9	2	1.6
2.6	2.1	5.5	2.5	2	1
2.5	2	5.6	2.4	2	1.2
2.5	1.2	6	3	1.8	1.2
2.2	1.6	5	2.2	1.8	1
2	1.7	5	2.4	1.5	1.1
2.5	2.6	7	3	1.5	2.5
2.6	2.1	6.6	2.7	2.1	1.8
3	1.9	6	2.5	2	1.5
2.8	3.2	7.3	3	2.1	2.2
2.6	2.6	7.6	2.8	2.1	2.7
2.2	2.3	6.8	2.8	2	2
2.6	2.5	7	3	1.8	2.2
2.5	2.5	7	3	2	2
2.5	2.9	6	2.6	1.9	1.5
3	2.5	7	2.9	2	2.1
2.5	2.7	7	2.6	2	2.4
2.7	2.3	7	3	2	2
2.5	2.6	7	3	1.5	2.5
2.6	2.1	6.6	2.7	2.1	1.8
3	1.9	6	2.5	2	1.5
2.8	3.2	7.3	3	2.1	2.2
2.6	2.6	7.6	2.8	2.1	2.7
2.2	2.3	6.8	2.8	2	2
2.6	2.5	7	3	1.8	2.2
2.5	2.5	7	3	2	2
2.5	2.9	6	2.6	1.9	1.5
3	2.5	7	2.9	2	2.1

2.5	2.7	7	2.6	2	2.4
2.7	2.3	7	3	2	2
2.5	2	5.5	2	1.5	2
2.5	1.9	7	2.8	1.9	2.3
2.9	2.1	6	2.5	2	1.5
1.9	1.8	5	2.2	1.4	1.4
2.5	2	5	2	1.5	1.5
2.4	1.8	5.9	2.5	2	1.4
3	1.6	6.4	2.7	2	1.7
2.6	2.1	6.6	2.7	2.1	1.8
3	1.9	6	2.5	2	1.5
2.8	3.2	7.3	3	2.1	2.2
2.6	2.6	7.6	2.8	2.1	2.7
2.2	2.3	6.8	2.8	2	2
2.6	2.5	7	3	1.8	2.2
2.4	1.8	5.9	2.5	2	1.4
3	1.6	6.4	2.7	2	1.7
2.6	2.1	6.6	2.7	2.1	1.8
3	1.9	6	2.5	2	1.5
2.8	3.2	7.3	3	2.1	2.2
2.6	2.6	7.6	2.8	2.1	2.7
2.2	2.3	6.8	2.8	2	2
2.6	2.5	7	3	1.8	2.2
2.5	2.5	7	3	2	2
2.5	2.9	6	2.6	1.9	1.5
3	2.5	7	2.9	2	2.1
2.5	2.7	7	2.6	2	2.4
2.5	2	5.5	2	1.5	2
2.9	2.1	6	2.5	2	1.5
1.9	1.8	5	2.2	1.4	1.4
2.5	2	5	2	1.5	1.5
2.5	2	5.5	2	1.5	2
2.9	2.1	6	2.5	2	1.5

1.9	1.8	5	2.2	1.4	1.4
2.5	2	5	2	1.5	1.5
2.5	2	5.5	2	1.5	2
2.9	2.1	6	2.5	2	1.5
1.9	1.8	5	2.2	1.4	1.4
2.5	2	5	2	1.5	1.5
2.2	1.6	5	2.2	1.8	1
2	1.7	5	2.4	1.5	1.1
2.2	1.6	5	2.2	1.8	1
2	1.7	5	2.4	1.5	1.1
2.2	1.6	5	2.2	1.8	1
2	1.7	5	2.4	1.5	1.1
2.2	1.6	5	2.2	1.8	1
2	1.7	5	2.4	1.5	1.1
2.2	1.6	5	2.2	1.8	1
2	1.7	5	2.4	1.5	1.1
2.2	1.6	5	2.2	1.8	1
2	1.7	5	2.4	1.5	1.1
2.5	2	5.5	2	1.5	2
2.9	2.1	6	2.5	2	1.5
1.9	1.8	5	2.2	1.4	1.4
2.5	2	5	2	1.5	1.5
2.5	2	5.5	2	1.5	2
2.9	2.1	6	2.5	2	1.5
1.9	1.8	5	2.2	1.4	1.4
2.5	2	5	2	1.5	1.5
2.2	1.6	5	2.2	1.8	1
2	1.7	5	2.4	1.5	1.1
2.2	1.6	5	2.2	1.8	1
2	1.7	5	2.4	1.5	1.1
2.2	1.6	5	2.2	1.8	1
2	1.7	5	2.4	1.5	1.1
2.2	1.6	5	2.2	1.8	1

2	1.7	5	2.4	1.5	1.1
2.2	1.6	5	2.2	1.8	1
2	1.7	5	2.4	1.5	1.1
2.2	1.6	5	2.2	1.8	1
2	1.7	5	2.4	1.5	1.1
